# Pyrrolysine supply and demand shapes the translational landscape in *Methanosarcina acetivorans*

**DOI:** 10.1073/pnas.2614934123

**Published:** 2026-10-08

**Authors:** Grace D. Britt, Daniel R. Utter, Yeonsoo Park, Victoria J. Orphan, Rodney Tollerson

**Affiliations:** ^a^ Department of Biological Sciences, Auburn University, Auburn, AL 36849; ^b^ Division of Biology and Biological Engineering, California Institute of Technology, Pasadena, CA 91125; ^c^ Division of Geological and Planetary Sciences, California Institute of Technology, Pasadena, CA 91125

**Keywords:** ribosome pausing, pyrrolysine, genetic code expansion, *Methanosarcina acetivorans*, tRNA charging

## Abstract

The amber stop codon (UAG) can encode for pyrrolysine (Pyl) or be read as a stop codon by methylamine-metabolizing organisms including methanogenic archaea. The fate of UAG is decided during the decoding step of translation by competition between the pyrrolysine-aminoacylated transfer RNA (Pyl-tRNA^Pyl^) and release factor. To further understand the consequences of pyrrolysine based genetic code expansion, we integrated RNA sequencing, tRNA charging analysis, and codon-resolved mono- and disome ribosome profiling in *Methanosarcina acetivorans*. During conditions of high pyrrolysine demand, we observed increased expression of the pyrrolysine biosynthetic operon with a concurrent increase in acylation of tRNA^Pyl^ and ribosome occupancy at UAG codons. During low pyrrolysine demand, the population of Pyl-tRNA^Pyl^ decreases and we observe a strong ribosome pausing signal during UAG decoding. We find that the dwell time on UAG codons is shorter during high demand, but ribosome collisions increase due to greater ribosome density on UAG-containing transcripts. Together, these results show how pyrrolysine demand modulates tRNA charging and controls elongation dynamics, clarifying the cellular consequences of decoding an ambiguous stop codon.

## Significance

Expansion of the genetic code through ambiguous stop codon usage is critical for increasing chemical diversity in many enzymes. For example, the UAG codon can be read as a termination codon or as pyrrolysine in *Methanosarcina*. Here, we study how the demand for pyrrolysine-containing enzymes modulates the capacity for stop codon readthrough in *Methanosarcina acetivorans*. In this study, we find that ribosome pausing at pyrrolysine codons is controlled by carbon source-dependent tRNA charging.

Stop codon suppression is found in all domains of life and can have many roles in cellular physiology, including diversifying proteomes, regulating enzyme stoichiometry, and insertion of unique amino acids with novel chemical moieties (1–4). One mechanism of stop codon readthrough is through genetic code expansion, where an aminoacyl tRNA pairs with a canonical stop codon (5, 6). Most documented events of stop codon suppression by a unique tRNA are performed by selenocysteine, which has a unique insertion element and translational elongation factor to prevent spurious stop codon readthrough at true ochre UGA nonsense codons (7). Selenocysteine recoding requires a SECIS RNA element and selenocysteine specific factors. Unlike selenocysteine, pyrrolysine-based suppression does not require an insertion element, and Pyl-tRNA^Pyl^ can be brought to the ribosome by canonical elongation factors (8, 9).

Pyrrolysine is found in both bacteria and archaea and is commonly tied to the consumption of methylamines (10–14). Pyrrolysine is biosynthesized by three enzymes, PylBCD, from two lysine precursor molecules. PylB converts lysine to 3-methylornithine, PylC ligates the methylornithine to the second lysine molecule, and PylD completes the synthesis of pyrrolysine by the oxidation of the product of PylC (15). Loss of *pylBCD* (pyrrolysine biosynthesis) or *pylTS* (pyrrolysine tRNA and synthetase) prevents growth on methylamines as the sole carbon source, but not when using alternative methylated carbon sources such as methanol, which requires methyltransferases that do not have in-frame UAG stop codons (16, 17). This is due to the methylamine methyltransferases being essential for methylamine utilization as the sole methanogenic carbon source in *M. acetivorans* (18, 19). The archaeal methylamine methyltransferases contain internal UAG stop codons which must be suppressed by tRNA^Pyl^ to produce full-length protein (20). To avoid nonsense suppression at true stop codons, methylamine-metabolizing methanogens have decreased the number of TAG codons in their genome. For example, ~5% of genes contain a TAG codon in genomes that putatively encode pyrrolysine machinery, while in organisms that do not use Pyl, the rate of TAG utilization increases to ~22% (8).

Members of the genus *Methanosarcina* generate methane from a variety of methylated carbon sources, including methanol, acetate, and methylamines (21, 22). Previous studies in *Methanosarcina acetivorans* have shown that when methylamines are present as the sole carbon source, transcription of corresponding methylamine methyltransferases increases (17, 19). This change in transcriptional activity leads to an increase in the demand for UAG stop codon suppression to synthesize full-length protein for methylamine-dependent metabolism. Currently, the role of methylamine methyltransferase demand on the UAG readthrough due to the increase in internal UAG-containing transcripts is unclear. When pyrrolysine demand is high, such as when the cell is using trimethylamine (TMA) as the sole carbon source, transcriptional and translational machinery must adapt. Transcriptionally, it has been recently observed that AmzR acts as a sensor for the presence of methylamines and represses expression of methylamine methyltransferases (23). Gene expression data have also shown that the pyrrolysine biosynthetic cluster is also upregulated during methylamine utilization (24). At the protein level, depletion of Pyl-tRNA^Pyl^ leads to an increase of truncated proteins from transcripts that have in-frame UAG codons (14). Furthermore, the UAG stop codon is recognized by the *Methanosarcina barkeri* release factors in vitro, suggesting that there is a competition between Pyl-tRNA^Pyl^ decoding and translation termination in vivo (25, 26). Since UAG can be read as both a stop and sense codon, we hypothesized that the interplay between a release factor mediated translation termination and Pyl-tRNA^Pyl^ stop codon suppression is likely impacted by the availability of aminoacylated tRNA^Pyl^. We also predicted that without rapid UAG decoding, ribosomes would pause or stall as what has been observed at standard termination codons in other organisms (27).

In this study, we use a multiomic approach to understand how pyrrolysine demand impacts the posttranscriptional landscape of the cell. We use *M. acetivorans*, a model methanogen that can utilize both methanol (low Pyl demand) and methylamines (high Pyl demand), to uncover the molecular dynamics underpinning pyrrolysine-based stop codon suppression (28). We predicted that there would be a carbon source-dependent control UAG decoding capacity in *M. acetivorans*. By comparing the transcriptional and translational profile of *M. acetivorans* during growth on methanol and TMA, we found widespread changes in the regulation of gene expression in the cell. Our results suggest that *pylT* transcription is consistent across carbon sources, while the tRNA charge state is dependent on the presence of methylamines. To determine the potential role of tRNA^Pyl^ in methanol conditions, we performed small RNA sequencing (RNA-seq) and found that tRNA^Pyl^ is processed into two major tRNA fragments of unknown function. We also compared nuclease treatments to develop a codon-level ribosome profiling protocol in *M. acetivorans*, revealing ribosome pausing and queueing at UAG when pyrrolysine demands were high. Additionally, we observed strong ribosome pausing events at GUA (valine) codons that are present in both methanol and methylamine conditions. By linking carbon source-dependent tRNA^Pyl^ charging to codon-resolved pausing and collisions, this study clarifies how pyrrolysine-based genetic code expansion alters the decoding landscape and highlights additional codon-specific elongation barriers beyond UAG.

## Results

### Expression of Pyrrolysine Biosynthesis Genes and the Charge State of tRNA^Pyl^ Are Dependent on the Presence of Methylamines.

**Fig. 1.**
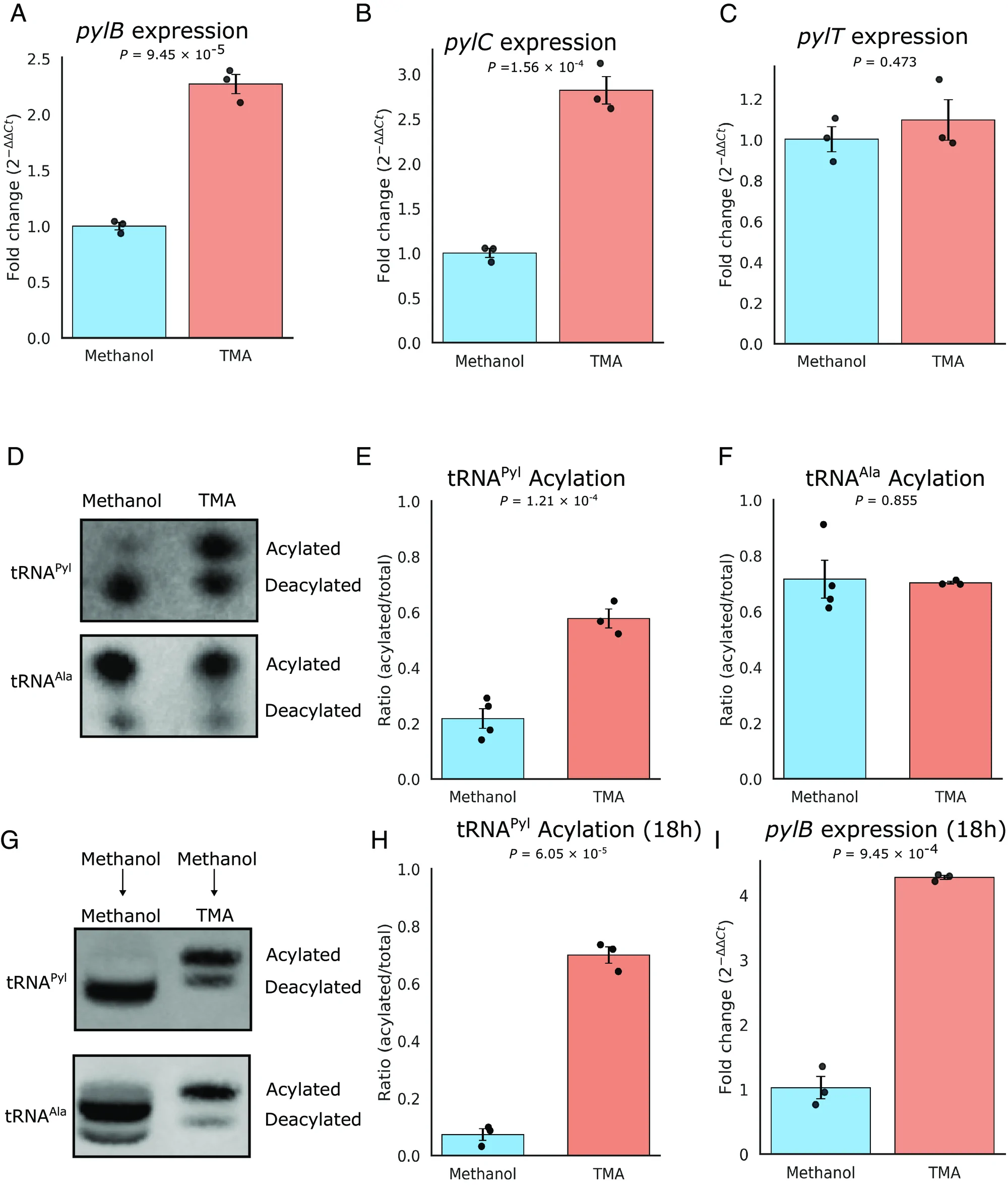
Pyrrolysine biosynthesis and tRNA^Pyl^ aminoacylation are correlated with pyrrolysine demand. (*A*) RT-qPCR data (n = 3 biological replicates of each condition) for *pylB* (*P* = 9.45 × 10^−5^), (*B*) *pylC* (*P* = 0.0001), and (*C*) *pylT* (*P* = 0.473) in both methanol and TMA conditions from total RNA of exponentially grown cells in triplicate. The normalization control for *pylBC* is *rpoB,* for *pylT* the control is *alaT.* Fold change was calculated using the ΔΔCt method, and the *P*-value was calculated using Student’s *t* test. (*D*) Representative northern blot of charging states of tRNA^Pyl^ (*Top*) and tRNA^Ala^ (*Bottom*). Total RNA was isolated from exponentially growing cells (n = 4 biological replicates for each condition) in methanol (lane 1) and TMA (lane 2), separated using acid-urea electrophoresis, and visualized using a probe specific to the tRNA. On each membrane, the top band is charged tRNA and the bottom band is uncharged tRNA. The membrane is imaged at IR700 for tRNA^Pyl^ and IR800 for tRNA^Ala^. (*E* and *F*) Quantification of the amount charged (n = 4 biological replicates of each condition). The ratio of acylated tRNA to total tRNA is calculated using ImageJ for methanol and TMA (Student’s *t* test, *P* = 1.21 × 10^−4^). Samples are compared to tRNA^Ala^ acylation (Student’s *t* test, *P* = 0.855). (*G*) Representative northern blot of charging states of tRNA^Pyl^. Total RNA was isolated from cells initially grown in methanol 18 h after the switchover (n = 3 biological replicates for each condition) to new media containing methanol (lane 1) or TMA (lane 2), separated using acid-urea electrophoresis, and visualized using a probe specific to the tRNA. On the membrane, the top band is charged tRNA and the bottom band is uncharged tRNA. The membrane is imaged at IR700. (*H*) Quantification of the amount charged (n = 3 biological replicates of each condition). The ratio of acylated tRNA^Pyl^ to total tRNA^Pyl^ is calculated using ImageJ for both methanol, on the left, and TMA, on the right (*P* = 6.06 × 10^−5^). (*I*) RT-qPCR data (n = 3 biological replicates of each condition) for *pylB* after 18 h switchover experiment from paired samples used for northern blot (*P* = 9.45 × 10^−4^).

To identify target genes that may have a role in adapting to pyrrolysine demand, we performed RNA-seq in *M. acetivorans* grown in 150 mM methanol (MeOH, low demand) or 50 mM trimethylamine (TMA, high demand). We compared the differentially expressed genes using DESeq2 and identified 23 genes that were downregulated and 30 that were upregulated in TMA compared to MeOH (|log_2_FC| ≥ 1 and adjusted *P*-value ≤ 0.05), including an observed log_2_FC of −5.5 for *mtaB* the methanol methyltransferase, and +5.5 for *mttB*, the trimethylamine methyltransferase (Dataset S1). We also observe a log_2_FC of 2.1 for *pylBCD* in TMA (*SI Appendix*, Fig. S1). These results are consistent with a recently published RNA-seq dataset that compares *pylBCD* levels under comparable conditions (17). We further confirmed this result using RT-qPCR to detect expression of *pylB* and *pylC*, which resulted in log_2_FC of 2.4 and 3.1, respectively (Fig. 1 *A* and *B*).

We hypothesized that the decrease in *pylBCD* expression would correlate with a lower percentage of Pyl-tRNA^Pyl^ when cells do not require pyrrolysine for growth (i.e., growth in MeOH), so we used northern blot to detect the state of the tRNA^Pyl^ pool in TMA and MeOH growth conditions. We separated tRNA by acylation state using acid urea gel electrophoresis and detected tRNA^Pyl^ and tRNA^Ala^ using IR700 (Pyl) and IR800 (Ala) probes complementary to the 5’ of the tRNA (Fig. 1*D*). We analyzed the intensity of the upper (acylated) band compared to the total intensity of the acylated and deacylated signal. We found that ~58% of tRNA^Pyl^ in TMA growth conditions is aminoacylated compared to ~21% in MeOH (Fig. 1 *D* and *E*). Our tRNA^Ala^ control was ~72% acylated in both conditions (Fig. 1 *D* and *F*). These results show that there is an increase in tRNA^Pyl^ charging during TMA growth that correlates with the increase in *pylBCD* expression. We also found that expression of *pylS* increased modestly but reproducibly in our RNA-seq dataset (Dataset S1, log_2_FC = 0.8, *P* = 1.99 × 10^−4^). Although this effect is below our threshold of |log_2_FC| ≥ 1, this may contribute to the observed differences in tRNA^Pyl^ aminoacylation.

To determine whether TMA alone was sufficient to induce tRNA^Pyl^ charging, we replaced the media of cells growing exponentially in methanol to media containing either methanol or TMA for one doubling time (18 h; Fig. 1*G*). We observed that the cells exposed to TMA were ~70% charged while the charging percent for methanol was ~7% (Fig. 1*H*). Our northern blots also suggest that tRNA^Pyl^ is well expressed in both MeOH and TMA conditions, so we determined the relative expression of *pylT* using RT-qPCR and we did not detect a significant difference between *pylT* abundance in either condition (Fig. 1*C*). Together, our results align with the prediction that translational machinery in *M. acetivorans* tunes its capacity for stop codon readthrough based on the demand for pyrrolysine.

### tRNA^Pyl^ Is Processed into Discrete Fragments.

**Fig. 2.**
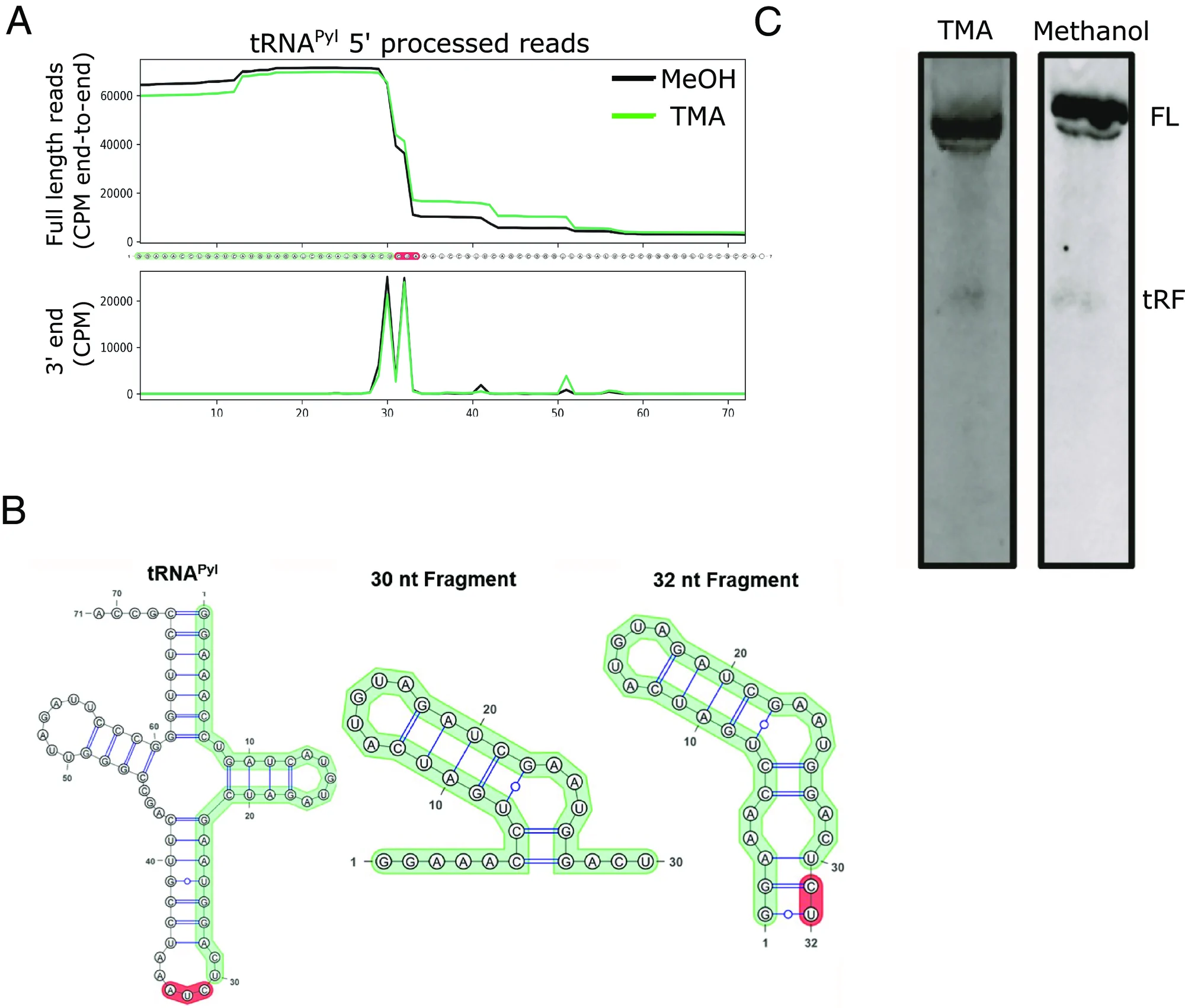
Pyrrolysine tRNA is processed into two tRNA fragments. (*A*) Counts per million (*y*-axis) coverage by end-to-end tRNA derived fragments mapping uniquely to tRNA^Pyl^ (*x*-axis). The green line represents TMA samples and the black line represents MeOH samples (n = 2). (*Bottom*) 3’ termini abundance for end-to-end samples. (*B*) Schematic representation of tRNA^Pyl^ made with RNA fold and VARNA with the anticodon highlighted in red and the most abundant putative tRNA fragment highlighted in green. Putative 30 nt tRNA^Pyl^ fragment with predicted folding using RNAfold is displayed in the middle and the 32 nt tRNA^Pyl^ fragment is displayed on the right with predicted folding using RNA fold that includes 2 nt of the anticodon highlighted in red. Double lines represent G:C base pairs, single lines represent A:U base pairs, and open circles denote noncanonical base pairs. The sequence for the *M. acetivorans* full-length tRNA^Pyl^ is 5’-GGAAACCUGAUCAUGUAGAUCGAAUGGACUCUAAAUCCGUUCAGCCGGGUUAGAUUCCCGGGUUUCCGCCA-3’. (*C*) Northern blot of full-length and processed tRNA^Pyl^_._ TMA is displayed on the left and methanol on the right.

Since our northern blots revealed a persistent pool tRNA^Pyl^ in both TMA and methanol conditions, we predicted that tRNA^Pyl^ may have a role beyond canonical amino acid transfer in both conditions. Mature tRNAs are often processed by regulated cleavage into tRNA-derived fragments (tRFs), so we performed small RNA-seq to determine whether tRNA^Pyl^ generates discrete fragments (29–32). We hypothesized that one explanation for maintaining a pool of tRNA^Pyl^ in both methanol and TMA conditions is that it becomes processed into fragments that may serve in functions beyond amino acid transfer. To determine putative tRFs, we used tRNAscan-SE to define the genomically encoded tRNAs and used those identifications to categorize the aligned small RNAs, using only the primary alignments (MAPQ ≥ 10). We analyzed the mapped reads to identify those with cleavage patterns that are indicative of site-specific tRF generation by calculating how often a specific fragment pattern occurs for a single isoacceptor. We observed two major tRNA^Pyl^ tRFs, one that is 30 nt and one that is 32 nt (Fig. 2*A*). Both fragments are generated from the 5’ end of the tRNA and make up 89% of the products made from tRNA^Pyl^. These tRFs also comprised 33% of the total 5’-containing mapped tRFs identified in our dataset. The termini of the tRFs were CU or CUCU, suggesting site-specificity for processing near the anticodon, where the final two bases (CU) of the 32 nt fragment contained the first two bases of the anticodon loop (Fig. 2*B*). We confirmed the presence of the tRNA^Pyl^-derived fragments from cells grown in MeOH and TMA using northern blotting with a probe annealing to the 5’ end of tRNA^Pyl^ (Fig. 2*C*). These data suggest that tRNA^Pyl^ is consistently processed at two specific locations near the anticodon loop and these fragments may have a potential role in *M. acetivorans* physiology.

### Development of Single Nucleotide Resolution Ribosome Profiling in *M. acetivorans*.

Our data and prior work by others suggest that demand for charged Pyl-tRNA^Pyl^ is regulated by methylamine availability (16, 17, 33). In other organisms, accumulation of deacylated tRNAs can slow decoding at codons that require the limiting isoacceptor, and reduced elongation rate can decrease ribosome processivity and promote ribosome collisions on highly translated mRNAs (34). Together with the dual role of UAG as a potential stop or sense codon, and the observed changes in charged tRNA pools, these observations led us to hypothesize that decoding at UAG is carbon source-dependent and may be slowed based upon the availability of Pyl-tRNA^Pyl^. We further hypothesized that slow decoding events could promote ribosome collisions on highly translated UAG-containing mRNAs during growth on TMA.

**Fig. 3.**
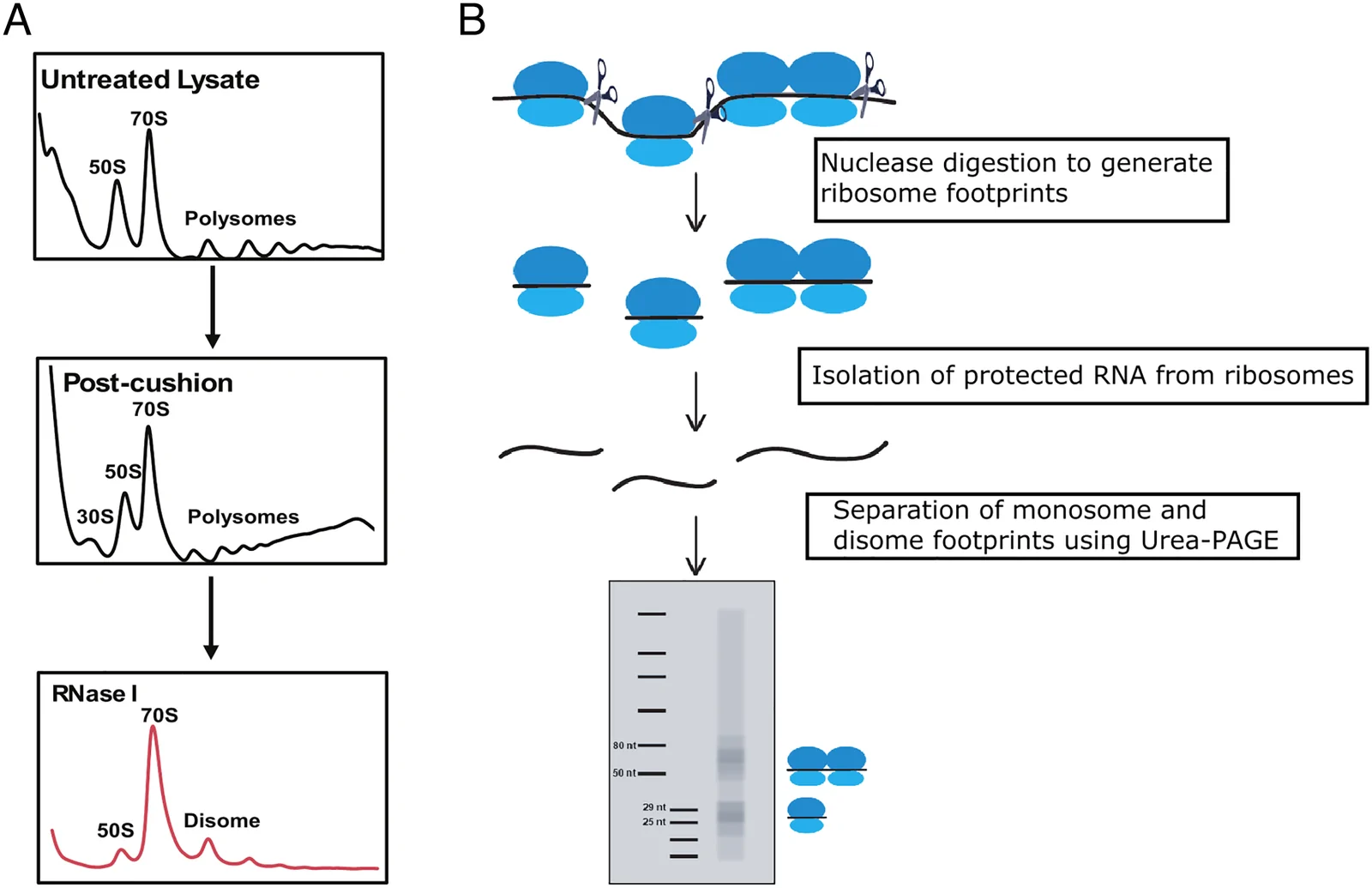
Schematic representation of ribosome profiling protocol. (*A*) Ribosome isolation and nuclease treatment by sucrose gradients. (*Top*) Untreated lysate displays subunits, monosome peak, and polysomes. (*Middle*) Cushioned lysate shows retention of polysomes. (*Bottom*) RNase I treated samples show a collapse of polysomes into monosome and disomes. (*B*) RPF isolation and size selection. Ribosomes are collapsed into monosomes and polysomes using nuclease (represented by scissors). Ribosome protected mRNA fragments are then isolated using phenol:chloroform isolation. Ribosome protected fragments are separated from other RNA fragments using Urea PAGE.

To identify codons associated with slow decoding in *M. acetivorans*, we developed a nucleotide-resolution ribosome profiling workflow (Fig. 3). Because nuclease choice can influence the position and heterogeneity of RPF termini, we compared RNase I_f_ (from here on referred to as RNase I) and nuclease P1 for footprint generation (35). We isolated and sequenced 25 to 29 nt (monosome) and 40 to 60 nt (disome) RPFs from samples treated with either RNase I or nuclease P1 (*SI Appendix*, Fig. S5).

**Fig. 4.**
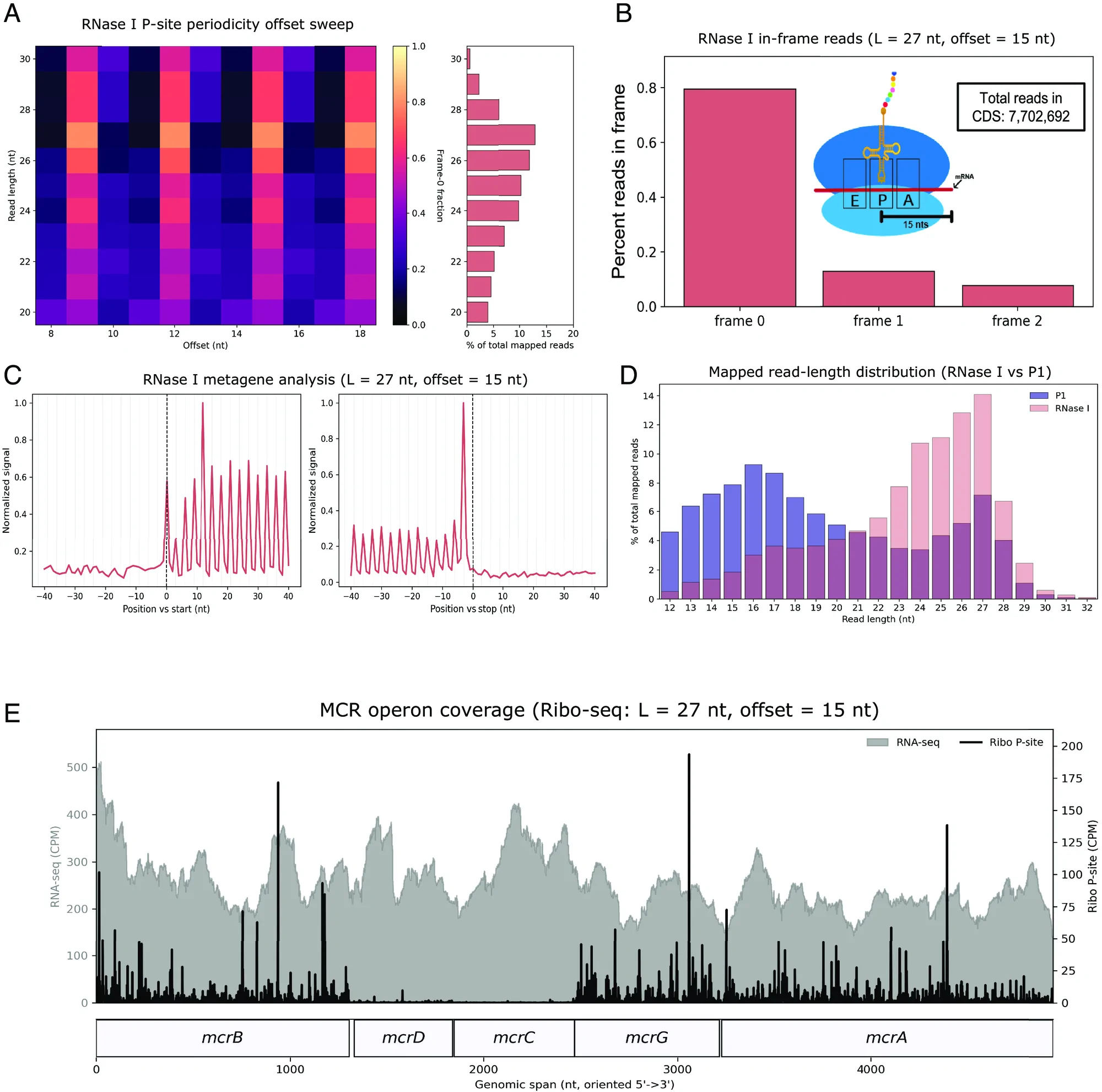
RNase I performs better than nuclease P1 for codon-resolved ribosome profiling in *M. acetivorans*. (*A*) RNase I heatmap displaying periodicity for specific lengths (*y*-axis) and offsets (*x*-axis). The color of each block corresponds to the color gradient on the right, representing different 3-nt periodicity percentages. On the right of the heat map is a bar graph of read length distribution. (*B*) Bar graph displaying the percentage of reads for RNase I samples in each frame for 27 nt length periodicity with an offset of 15 nt from the 3’ end. (*C*) Metagene plots displaying the start (*Left*) and stop (*Right*) codon for methanol RNase I RPFs using the P-site. The graphs display the 27 nt RPFs with an offset of 15 nt from the 3’ end. (*D*) Mapped read length distribution for P1 samples (blue) and RNase I samples (red). The *x*-axis displays length in nucleotides, and the *y*-axis displays the percentage of reads at the specific length. (*E*) Representative coverage graph for the MCR operon displaying the ribosome P-site density in black and RNA-seq coverage of each gene displayed in gray in the background.

A critical quality control metric for ribosome profiling is 3-nt periodicity, which reflects proper capture of actively elongating ribosomes and reduces the likelihood of technical artifacts in data analysis (35–42). With high 3-nt periodicity, it becomes possible to confidently determine the location of the ribosomal A, P, and E-sites which is essential for assessing decoding rates and ribosome dwell times on specific codons. In both datasets, 27 nt footprints exhibited the strongest 3-nt periodicity when calculating the position of the ribosomal P-site in relation to the 3’ end of each read, with RNase I showing enrichment for in-frame 27 nt footprints and more consistent termini (Fig. 4 *A*–*D*).

### Translational Regulation May Contribute to MCR Subunit Stoichiometry.

Having established that RNase I treatment produced RPFs with strong three-nucleotide periodicity, we next asked whether the resulting profiles could detect biologically meaningful variation in translation across a well-characterized operon. We selected the *mcrBDCGA* operon as an independent test case because methyl-coenzyme M reductase (MCR) is highly expressed in methanogens, providing sufficient coverage for gene-level analysis, and because the operon encodes proteins with distinct stoichiometric and functional requirements. Importantly, the MCR proteins do not contain pyrrolysine-dependent UAG codons. Instead, our analysis provided an orthogonal assessment of whether our profiling workflow could distinguish translational regulation among genes encoded on the same polycistronic transcript. MCR is an α_2_β_2_γ_2_ heterohexamer composed of the catalytic McrA, McrB, and McrG subunits (43, 44). McrC and McrD are conserved accessory proteins involved in MCR activation and assembly, respectively (45, 46). Recent studies have suggested that McrD is required at a lower stoichiometry than McrABG and can interact with multiple MCR complexes (46). Because these proteins are encoded within the same operon but are expected to have different cellular requirements, we hypothesized that translation of the accessory proteins would be lower than translation of the catalytic subunits. Comparison of CPM-normalized RNA-seq coverage and 27 nt RPF density revealed relatively lower ribosome occupancy over *mcrC* and *mcrD* than over *mcrABG* (Fig. 4*E*). These results demonstrate that our ribosome-profiling workflow can detect biologically interpretable differences in translation across a highly expressed polycistronic transcript, supporting its subsequent application to codon-resolved analysis of UAG decoding.

### UAG Codons Lead to Increased Ribosome Dwell Time.

The impact of pyrrolysine codon translation, due to its ambiguous status as both a stop and sense codon, at the level of elongation is currently uncertain. We hypothesized that the increase in demand for Pyl-tRNA^Pyl^ by the translational machinery would change the ribosome pausing landscape in *M. acetivorans*.

**Fig. 5.**
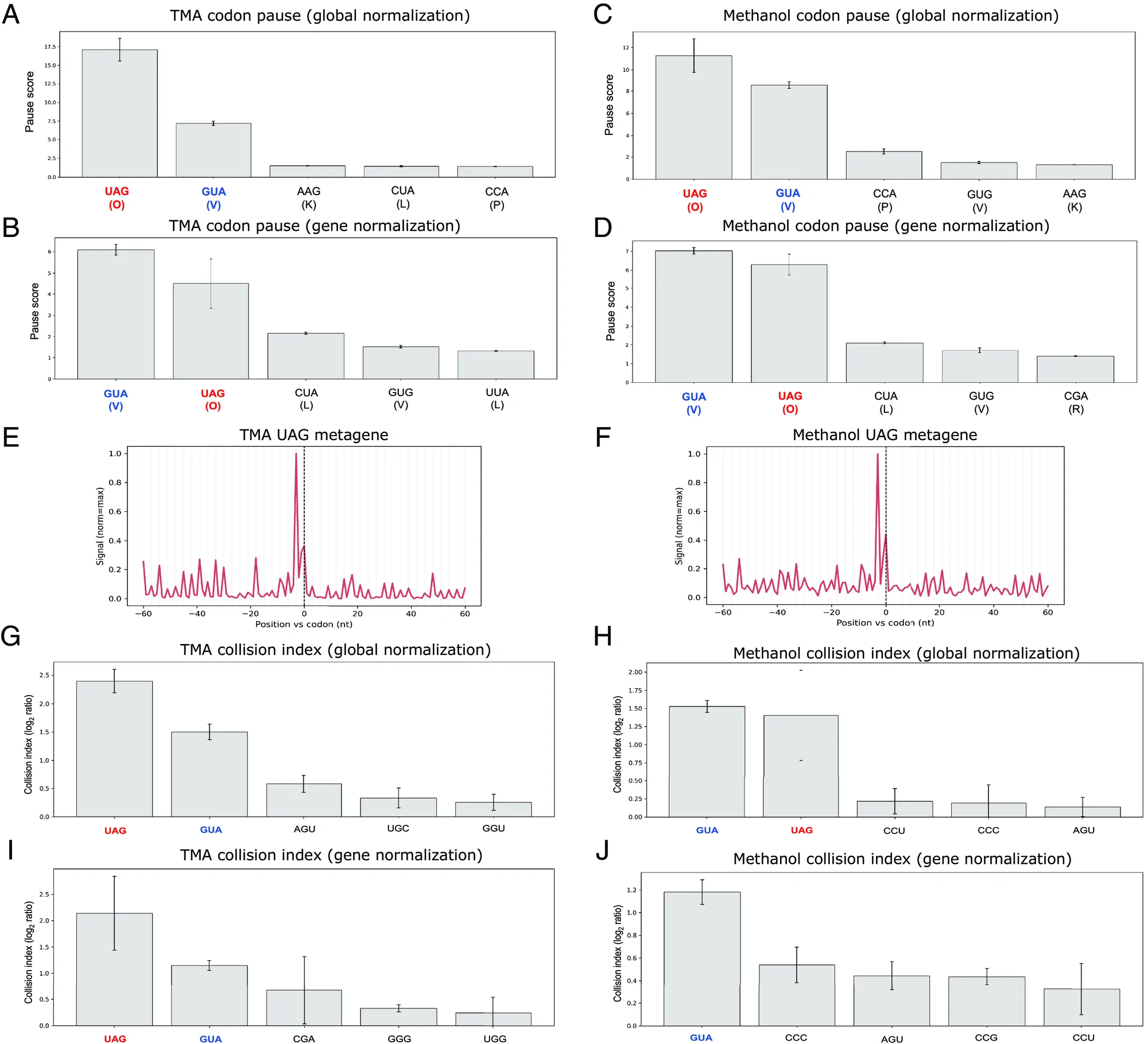
Codon-level ribosome profiling in *M. acetivorans* reveals pausing at pyrrolysine codons. Top five codon pauses for TMA monosome footprints (27 nt) using the global ratio (*A*) and gene normalized (*B*) methods. Pause score is calculated by A-site coverage of each specific codon. Top five codon pauses for MeOH monosome footprints (27 nt) using the global ratio (*C*) and gene normalized (*D*) methods. Pause score is calculated by A-site coverage of each specific codon. Metagene analysis using the UAG codon as the 0 site in both TMA (*E*) and methanol (*F*) conditions. All samples are averaged together for a single score and use the P-site as the peak. Collision index graph calculated using log_2_ ratio of disome pausing to monosome pause using global (*G* and *H*) and gene-based (*I* and *J*) normalization.

We tested this by calculating the pause score in MeOH and TMA conditions using our 27 nt (monosome) RPFs. We used two normalization methods to capture both translational demand (global normalization) and pausing intrinsic to the codon itself (gene normalization). Global normalization calculates the difference between expected codon presence based on RNA-seq and its presence in the A-site of the ribosome. Gene normalization decreases the sensitivity to highly translated genes by internally controlling for total ribosome occupancy in the coding sequence. In our monosomes, we observed the greatest ribosome occupancy when a pyrrolysine codon is positioned in the A-site in both MeOH and TMA-grown samples. The global pause score was greater in TMA grown cells than those in MeOH, this contrasts with a higher gene normalized pause score in MeOH than in TMA (Fig. 5 *A*–*D*). These results suggest that demand for Pyl-tRNA^Pyl^ is higher in TMA, but the ribosome dwell time at UAG in MeOH growth is longer. We used metagene analysis to visualize the impact of ribosome pausing at UAG on the surrounding codons. We observe a slight decrease in ribosome occupancy on codons immediately following UAG compared to those upstream in both TMA and MeOH conditions (Fig. 5 *E* and *F*). These results are consistent with behavior of UAG as both a stop and sense codon.

To connect the aggregate UAG pause signal to established Pyl-dependent genes, we examined ribosome occupancy across the monomethylamine, dimethylamine, and trimethylamine methyltransferase genes. The internal UAG codon in *mtmB* was associated with a prominent local peak in P-site occupancy, providing a gene-level example of pausing at a physiologically required pyrrolysine insertion site (*SI Appendix*, Fig. S7*A*). In contrast, the internal UAG codons in *mtbB* and *mttB* did not show comparably distinct peaks in the corresponding whole-gene profiles (*SI Appendix*, Fig. S7 *B* and *C*). These results indicate that the prominence of UAG-associated pausing varies among Pyl-dependent transcripts.

To determine whether slow decoding at UAG is accompanied by ribosome collisions, we also performed disome profiling. Disome profiling captures RNA protected by two ribosomes in close enough proximity that the nuclease cannot efficiently cleave between them (36, 47–49). We found that the 54 and 57 nt RPFs from disomes had the strongest periodicity for codon analysis. The propensity of a paused ribosome to cause a collision was calculated by comparing the pause score for monosomes at a specific codon to the disome pause score. To quantify collision propensity directly, we computed the collision index, defined as the log_2_ ratio of disome to monosome pause scores, for each codon. We found that TMA growth conditions led to higher rates of observable collisions compared to MeOH (Fig. 5 *G* and *H*). We also find that when using within-gene normalization, the collisions in the MeOH-grown cells are further suppressed (Fig. 5 *I* and *J*).

These results suggest that while demand for Pyl-tRNA^Pyl^ is higher in TMA, the ribosome dwell time at UAG in MeOH growth is longer. Because global normalization retains the contribution of highly translated UAG-containing genes, the elevated global score in TMA is consistent with increased translational demand for Pyl-containing proteins. In contrast, the elevated gene-normalized score in methanol indicates greater UAG enrichment relative to ribosome occupancy within individual coding sequences, consistent with a longer dwell time when the estimated aminoacylated fraction of tRNA^Pyl^ is low.

### Single Codon-Level Pausing Identifies the GUA Codon as a Slow Decoding Event.

**Fig. 6.**
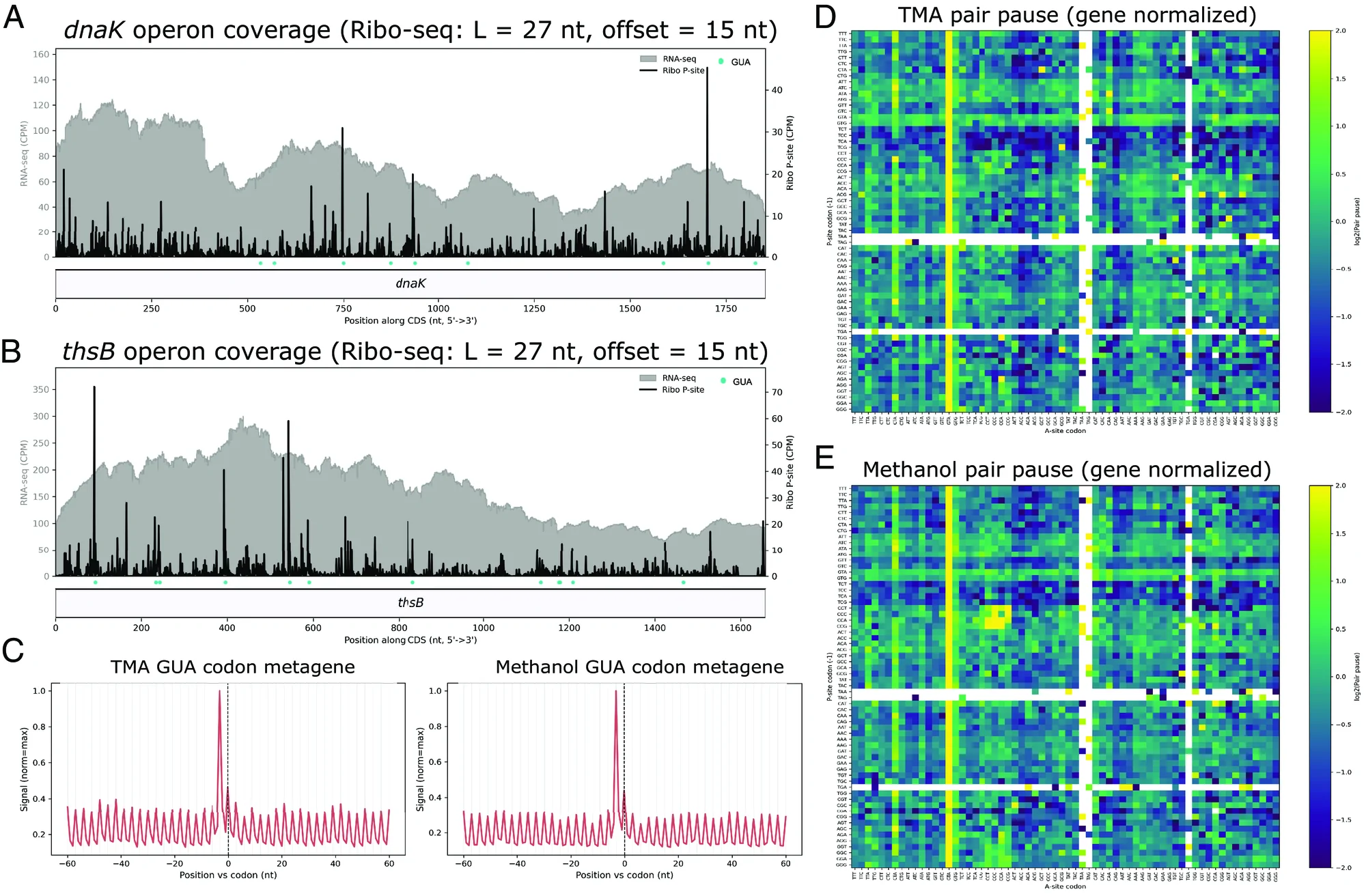
Analysis of GUA pausing in TMA and MeOH. Coverage graph for *dnaK* (*A*) and *thsB* (*B*) displaying the ribosome density in black based off of the P-site for each footprints and RNA-seq coverage of each gene displayed in gray in the background. GUA codons are denoted by blue points. (*C*) Metagene analysis using the GUA codon as the 0 site in both TMA (*Left*) and MeOH (*Right*) conditions. All samples are averaged together for a single score and the P-site is distinguished as a peak. The *y*-axis is normalized to the strongest signal. Pair pause heat map showing the TMA (*D*) and MeOH (*E*) monosome pause score of the A-site codon (*x*-axis) dependent on the P-site codon (*y*-axis).

Surprisingly, we also observed a strong pause at valine GUA codons, suggesting that ribosome processivity is reduced at GUA-decoded sites (Fig. 6). Notably, the GUA pause signal was consistent across normalization strategies, arguing against an explanation driven solely by a small set of highly expressed genes. The total valine signal mapped predominantly to the GUA valine codon (Fig. 5 *A*–*D*). Other valine codons did not show comparably elevated pause scores, indicating that the observed pausing is not a property of valine incorporation but instead reflects codon-specific decoding dynamics (*SI Appendix*, Fig. S8). We also observed increased GUA occupancy in disomes across conditions, indicating that slowed decoding at GUA may promote ribosome collisions (Fig. 5 *G*–*J* and *SI Appendix*, Fig. S9). We calculated the collision index using our codon resolved metrics and found that the GUA codon led to a similar collision index as what we observed when we aggregate all valine codons, suggesting that the pausing and collision signals are predominantly due to slow decoding at GUA.

We found that in the highest expressed genes, the GUA codon leads to high ribosome occupancy but not clear asymmetry of translation across the transcript (Fig. 6 *A* and *B*). We used a codon metagene analysis to determine the ribosome occupancy on the surrounding codons and observe a strong peak when GUA is in the A-site in both MeOH and TMA conditions (Fig. 6*C*). This pause does not change the occupancy of downstream codons, suggesting that the slow decoding event does not impact overall processivity.

The surrounding genetic context of a codon can influence the rate of decoding and peptide bond formation (41, 42, 50–53). To assess the contribution of the P-site codon on pausing in *M. acetivorans*, we generated a 64 × 64 codon-pair matrix (Fig. 6 *D* and *E*). This matrix displays the gene normalized pause score of each codon in the A-site depending upon the identity of the P-site codon. We find that in both monosomes and disomes the GUA codon causes strong pausing independent of the P-site codon. In our monosome samples, the GUA codon does not lead to increased ribosome occupancy when in the P-site, suggesting a decrease in decoding speed instead of a decrease in the rate of peptidyl transfer. In disome footprints, GUA-associated pausing occurs with the GUA codon positioned in either the A site or the P site, which may reflect the spatial organization of queued ribosomes around a strong pause site.

## Discussion

Genetic code expansion increases the opportunities to regulate gene expression at the posttranscriptional level. Pyrrolysine translation requires a novel biosynthetic pathway, tRNA, and aminoacyl tRNA synthetase to be efficiently incorporated into proteins. In this study, we find that ribosome pausing at UAG codons is ubiquitous, but when demand for Pyl-tRNA^Pyl^ is low, the ratio of charged to total tRNA^Pyl^ decreases significantly and correlates with longer dwell times for ribosomes with UAG in the A-site. We also observe unexpected pausing events at GUA valine codons, suggesting that decoding of GUA is intrinsically slow compared to other sense codons. This study clarifies the relationship between translational demand, tRNA^Pyl^ aminoacylation state, and ribosome processivity.

### Role of tRNA^Pyl^ in Both Methanol and TMA Conditions.

Our northern blot and qPCR data show that there is a large pool of uncharged tRNA^Pyl^ in cells independent of pyrrolysine demand (Fig. 1), while the amount of charged tRNA^Pyl^ shifts with the expression of *pylBCD*. The basis of the differential charging of tRNA^Pyl^ in the methanol and TMA conditions is currently unknown. This result may be due to changes in precursor availability, the enzymatic activity of PylBCD and PylRS, or the rate of Pyl-tRNA^Pyl^ consumption by translation. Further studies in the metabolite profile and enzymatic activity of Pyl-related biosynthesis and charging machinery in *M. acetivorans* will be necessary to identify the mechanism of the dynamic tRNA^Pyl^ charging.

We further explored a potential role of tRNA^Pyl^ using small RNA-seq to search for putative tRNA fragments. We identified two major tRFs that were abundant in both conditions that are strongly enriched in our small RNA-seq dataset (Fig. 2*A*). We find that these fragments both have identical last two bases at the 3’ ends (CU) which is consistent with site-specific processing (Fig. 2*B*). The 3’ end of the tRFs are also proximal to the anticodon, a common region for tRF processing (54). The 30 nt and 32 nt fragments are predicted to fold differently, though it is uncertain if the two fragments have different roles in the cell. We are not certain of the function that these tRFs may have in *M. acetivorans* physiology, but the generation of these fragments may highlight an additional role of tRNA^Pyl^ in both methanol and TMA conditions, though the role of these fragments remains unknown.

### Development of Codon-Resolved Ribosome Profiling.

In previous ribosome profiling protocols, antibiotics have been used at different steps to inhibit translation elongation. This enriches for polysomes (indicative of high ribosome density on an mRNA region) which are critical for ensuring that actively translating ribosomes are being captured for ribosome-protected fragment (RPF) generation, but the use of some translational inhibitors before cell lysis can lock the ribosome in conformations that are not representative of the active translational state (55–57). For this reason, we omit antibiotic use from our protocol to avoid misinterpretation of pause site location. Instead, we use rapid chilling by pouring the cells over loosely packed ice prior to centrifugation. After centrifugation, we immediately flash freeze the sample in liquid nitrogen. We found that this technique retains polysomes in *M. acetivorans* without the requirement of antibiotics.

We found that by removing nonprotected RNA using sucrose cushions and filtration, we preserved polysomes while increasing the yield of RPFs. The sucrose cushion reduces contamination from small RNAs that may be the size of RPFs and we found that it decreases the amount of nuclease required for efficient of polysome collapse compared to digesting lysate. After nuclease treatment, we removed RNA that was digested to RPF sizes without being protected by a ribosome by filtration using a 100 kDa MWCO filter. Ribosome profiling protocols have differed in the way that RPFs are generated and we find that this method balances stringency for RPFs and yield.

In our dataset, we find that the 27 nt RPFs contain reads with the greatest 3-nt periodicity independent of the nuclease choice (Fig. 4 and *SI Appendix*, Fig. S6). This is consistent with previously reported RPF length in *Haloferax volcanii* and our analysis of recently published ribosome profiling data in *Methanosarcina mazei* (50, 58). This contrasts with RPF lengths that have been observed in bacteria and eukaryotes which range from 24 nt in *Escherichia coli* to 28 nt in *Saccharomyces cerevisiae* (42, 47). Archaeal ribosomes may have a conserved size when actively elongating and the role between the physical conformation of the ribosome and steps in the elongation cycle in archaea require further study.

### Translational Demand Leads to Different Pause Landscapes in *M. acetivorans*.

Stop codon suppression by pyrrolysine has been predicted to have a role in metabolism of a growing number of environmentally and evolutionarily relevant organisms (17, 59). Recent studies have shown that in the absence of pyrrolysine biosynthesis, UAG-containing proteins become truncated due to its ambiguous sense/stop codon usage (17). It was also predicted that pyrrolysine supply in TMA conditions is lower than demand. Our data support a model that the demand for Pyl-tRNA^Pyl^ reprograms posttranscriptional information processing in *M. acetivorans*. The increased abundance and translation of UAG-containing methylamine-utilization transcripts during TMA growth coincided with greater global ribosome occupancy and an increased aggregate collision signal at UAG codons (Fig. 5 *G*–*J*). This is consistent with an increase in ribosome traffic on UAG-containing mRNAs in the TMA growth condition. In MeOH growth, the dwell time at UAG is longer, potentially due to a lack of charged tRNA^Pyl^, but this dwell does not lead to collisions because of the lower ribosome abundance on the transcripts (Fig. 5 *H* and *J*). This highlights the interplay between demand for Pyl-containing proteins and efficiency of stop codon readthrough. Though the efficiency of readthrough increased in TMA based on our pausing data, the occupancy of ribosomal A-sites on UAG codons is high, leading to collisions based on translational demand. In MeOH, the charge ratio is low for Pyl-tRNA^Pyl^, corresponding with demand, and this leads to longer decoding times without the risk of collisions.

Gene-level analysis further demonstrated that UAG-associated ribosome occupancy is transcript dependent. Among the Pyl-dependent methylamine methyltransferases examined, *mtmB* showed the clearest local P-site enrichment at its internal UAG codon. The corresponding UAG sites in *mtbB* and *mttB* were less prominent, and their profiles contained stronger GUA-associated pauses (*SI Appendix*, Fig. S7). These differences could reflect variation in ribosome flux, local codon context, mRNA structure, or other transcript-specific features. Thus, although the aggregate analysis identifies UAG as a major source of ribosome pausing, individual Pyl-dependent transcripts do not exhibit identical occupancy patterns.

Together, our data reveal the molecular consequences of genetic code expansion by pyrrolysine in *M. acetivorans*. Since pyrrolysine is not required for growth in MeOH conditions, the cell decreases its biosynthesis by downregulating *pylBCD*, likely leading to a depleted pool of the amino acid pyrrolysine. Lowered levels of pyrrolysine correlate with decreased tRNA^Pyl^ aminoacylation and increased ribosome decoding time on UAG-containing transcripts. During high pyrrolysine demand, such as when the cell is growing with TMA, biosynthesis of pyrrolysine increases as well as the ratio of aminoacylated tRNA^Pyl^. This increased ratio does not prevent ribosome pausing at UAG codons, instead the increased demand for pyrrolysine-containing proteins leads to ribosome collisions proximal to the UAG codon. Together, these results establish how pyrrolysine-dependent decoding shapes elongation dynamics and ribosome behavior in *M. acetivorans*, revealing broader principles by which genetic code expansion rewires translation.

## Materials and Methods

### Cell Growth.

*M. acetivorans* C2A were grown in high salt media containing either 150 mM methanol or 50 mM trimethylamine (TMA) (22). Cells were grown in anoxic conditions (80% N_2_/20% CO_2_ headspace) at 37 °C using stoppered 134 mL serum vials.

### tRNA Northern Blot.

Cells from each growth condition were collected as four independent biological replicates. Methanol- and TMA-grown samples were harvested over ice and processed in parallel using identical extraction, electrophoresis, transfer, hybridization, and imaging conditions. Cell pellets were resuspended in TRI Reagent (Zymo), flash frozen in liquid nitrogen, and lysed by bead beating. Total RNA was isolated by standard phenol chloroform extraction and precipitation. For each biological replicate, an aliquot of total RNA was deliberately deacylated to establish the migration position of uncharged tRNA (*SI Appendix*, Fig. S2). RNA was transferred to a membrane, dried, and prehybridized. Hybridization buffer was exchanged and pyrrolysine and alanine tRNA probes were added and incubated overnight. After hybridization, the membrane was washed and imaged. The aminoacylated fraction was estimated by dividing the background-corrected intensity of the upper band by the combined background-corrected intensities of the upper and lower bands. Statistical analysis was performed using a two-sided unpaired Student’s *t* test.

The same protocol was used for tRNA fragments except for the separation of small RNA from total RNA. Additionally, the RNA was run on a denaturing polyacrylamide gel instead of an acid gel.

For the carbon source switchover assay, cells were grown to exponential phase and harvested by centrifugation. The supernatant was removed and the cells were resuspended in basal growth media with no carbon source. The resuspended culture was put into new tubes with either methanol or TMA. These cultures were grown for 18 h, then harvested by centrifugation. The pellet was then resuspended in TRI Reagent and processed identically to the initial northern blot assays.

### RT-qPCR.

Total RNA was isolated from *M. acetivorans* lysate and treated with DNase I. The RNA was then diluted, and RT-qPCR was performed on the Applied Biosystems QuantStudio® 6 using the Luna® Universal Probe One-Step RT-qPCR Kit (NEB). Relative expression was calculated using the ΔΔCt method using reference genes (*SI Appendix*, Table S1) as the internal control.

### RNA-Seq.

*M. acetivorans* was grown, harvested, and lysed as previously described. The RNA was extracted from the lysate with DNase I treatment. Purified total RNA was rRNA depleted and RNA libraries were prepared and quantified with a Qubit. cDNA was then pooled to 10 nM and sequenced by SeqCenter (Pittsburgh, PA).

### Ribosome Profiling.

*M. acetivorans* was grown as previously described and harvested by centrifugation over ice and the pellets flash-frozen in liquid nitrogen until processing. Frozen cell pellets were lysed by bead beating in lysis buffer. Lysate was clarified and layered over a 60% sucrose cushion then pelleted by ultracentrifugation. The ribosome pellets were resuspended in digestion buffer. Each sample was split and treated with RNase If and P1 (NEB). The ribosomes were isolated using spin columns followed by removal of protein, end repair, and precipitation of the ribosome protected fragments. The RNA was size selected on a denaturing polyacrylamide gel. Monosome and disome fragments were extracted, converted to cDNA, and libraries were prepared and sequenced on an Illumina platform.

### Small RNA.

*M. acetivorans* was grown and harvested as previously described. The cell pellets were resuspended TRIreagent (Zymo) and the RNA was extracted using standard phenol chloroform, followed by DNase I treatment. Small RNA was separated from total RNA and the ends were repaired. Purified small RNA was then run on a denaturing polyacrylamide gel, size selected, and precipitated. The RNA was converted to cDNA, and libraries were prepared and sequenced on an Illumina platform.

### Preprocessing of Sequencing Samples.

Adapters were removed using cutadapt v5.2, allowing for 1 mismatch and keeping only reads with Q ≥ 30. Reads for ribo-seq were then filtered by length and non-coding RNA removal (for RNA-seq and ribo-seq samples) was performed using Bowtie2 v2.5.4. Reads that passed filter were then mapped using Bowtie2 to the reference genome, allowing for 1 mismatch and discarding any multimapping reads. The resulting files were sorted and indexed using samtools v1.22.1 and counted using featureCounts (subread v2.1.1).

### Differential Expression Analysis.

Differential expression analysis was performed with DESeq2 using a negative binomial generalized linear model and Wald tests for the specified contrast (60). Log_2_FC was shrunk using lfcShrink with the apeglm method to provide stabilized effect-size estimates for ranking and visualization. Genes were considered differentially expressed if *P*_adj_ ≤ 0.05 and |Log_2_FC| ≥ 1.

### Ribosome Profiling Data Analysis.

Ribosome profiling data were analyzed using a combination of previously published methods (36, 47, 50, 61–63).To adapt these protocols to our dataset, we used Microsoft 365 Copilot as a coding assistant to generate initial drafts of selected scripts (e.g., boilerplate structure, helper functions, and visualization routines) based on author-specified requirements and peer-reviewed reference code. Copilot assistance was limited to code scaffolding for the offset sweep, pause score, and collision index pipelines; analytic decisions, parameter selection, and interpretation were determined by the authors. All generated code underwent manual review, revision, and testing, and was validated against published datasets and expected outputs (50, 58). The authors take full responsibility for the final codebase and all analyses.

### Periodicity Offset Sweep.

To identify the read length and end-based offset that maximized 3-nt periodicity, we pooled uniquely mapped RPF and evaluated candidate read lengths and offsets. For each read length *L* and offset *O*, we defined an end coordinate for each primary alignment (3′-end by default), then inferred a P-site position by shifting that end coordinate by *O* nucleotides in a strand-aware manner. For each (*L*, *O*) pair, we assigned each retained P-site to a reading frame relative to the CDS start (frame = 0/1/2, strand-aware) and calculated the fraction of CDS-assigned reads in-frame 0. The optimal (*L*, *O*) combination was selected as the pair that maximized the frame-0 fraction.

### Pause Score.

We calculated the pause score for each condition by aggregating the reads from biological replicates (n = 3) grown in either MeOH or TMA. The A-site codon was identified by determining P-site coordinate using the 27 nt read length with an offset of 15 nt. The final 5 codons (15 nt) and any in-frame premature TAA and TGA stop codons were excluded. Biological replicates were processed independently then summarized as the mean pause score across replicates. Two complementary pause score normalization metrics were computed from A-site assignments: gene normalized, site-weighted pause score and global normalization, expression weighted.

For each codon or amino acid x in gene g, we defined a per-gene pause enrichment as

[1] $$P_{g,x}=\frac{R_{g,x}/R_{g}}{N_{g,x}/L_{g}},$$

where $R_{g,x}$ is the number of ribosome footprints assigned to x in gene g, $R_{g}$ is the total number of assigned footprints in that gene, $N_{g,x}$ is the number of occurrences of x in the analyzed coding region, and $L_{g}$ is the total number of analyzed codons in that region (Eq. **1**).

The gene-normalized pause score was calculated as an equal-weight average across genes containing x (Eq. **2**)

[2] $${\mathrm{PauseScore}}_{x}^{\mathrm{gene}\text{-}\mathrm{norm}}=\frac{1}{\left|G_{x}\right|}\sum_{g\in G_{x}}P_{g,x}.$$

The global pause score was calculated as

[3] $${\mathrm{PauseScore}}_{x}^{\mathrm{global}}=\frac{R_{x}/\sum_{y}R_{y}}{E_{x}},$$

where $R_{x}/\sum_{y}R_{y}$ is the observed global frequency of x and $E_{x}$ is its expected frequency in the analyzed coding regions (Eq. **3**).

### Collision Index.

The collision index was calculated by taking the ratio of the pause score (paired by condition and normalization method) from monosome reads (length = 27 nt, offset = 15 nt) and disome (length = 54 nt and 57 nt, offset = 15 nt). Variance was calculated by SEM.

### Disclaimer.

This report was prepared as an account of work sponsored by an agency of the United States Government. Neither the United States Government nor any agency thereof, nor any of their employees, makes any warranty, express or implied, or assumes any legal liability or responsibility for the accuracy, completeness, or usefulness of any information, apparatus, product, or process disclosed, or represents that its use would not infringe privately owned rights. Reference herein to any specific commercial product, process, or service by trade name, trademark, manufacturer, or otherwise does not necessarily constitute or imply its endorsement, recommendation, or favoring by the United States Government or any agency thereof. The views and opinions of authors expressed herein do not necessarily state or reflect those of the United States Government or any agency thereof.

## Supplementary Material

Appendix 01 (PDF)

Dataset S01 (XLSX)

Dataset S02 (XLSX)

Dataset S03 (XLSX)

## Data Availability

Raw Illumina reads for RNA-seq data can be found in SRA project PRJNA1135498 (64). Ribosome profiling and small RNA seq can be found in SRA project PRJNA1458217 (65). Software used for this paper can be found in the Github repository: https://github.com/rtollerson/britt_2026 (66). All other data are included in the manuscript and/or supporting information.
