# Supplementary material for: Pyrrolysine supply and demand shapes the translational landscape in Methanosarcina acetivorans
Source: Proc Natl Acad Sci U S A. 2026 Oct 8;123(41):e2614934123. doi: 10.1073/pnas.2614934123 (PMC13663744; doi:10.1073/pnas.2614934123)
Supplement: Supplementary file 1 [file pnas.2614934123.sapp.pdf]

PNAS

Supporting Information for

Pyrrolysine supply and demand shapes the translational landscape in *Methanosarcina acetivorans*

Grace D. Britt, Daniel R. Utter, Yeonsoo Park, Victoria J. Orphan, Rodney Tollerson II*

*To whom correspondence should be addressed:

Rodney Tollerson II: tollerson@auburn.edu

This PDF file includes:

- Extended Materials and Methods
- Figures S1 to S9
- Table S1
- Legends for Datasets S1 to S3
- SI References

Other supporting materials for this manuscript include the following:

- Datasets S1 to S3

Methods

Cell growth

Methanosarcina acetivorans C2A (DSM 2834, received from Leibniz Institute DSMZ) were grown in high salt media (Per liter: 0.34 g KCl, 4 g $MgCl_2 \times 6H_2O$, 3.45 g $MgSO_4 \times 7H_2O$, 0.25 g NH_4Cl, 0.14 g $CaCl_2 \times 2\ H_2O$, 0.14 g K_2HPO_4, 18 g NaCl, 5 g $NaHCO_3$, 1 g CH_3COONa, 0.5 g cysteine × H_2O, 500 μL 0.1% resazurin, 10 mL Wolin's mineral solution, 1 mL Wolin's vitamin solution, and 1 mL of 0.5g/mL $Na_2S \times 9H_2O$) containing either 150 mM methanol or 50 mM trimethylamine (TMA) (1). Cells were grown in anoxic conditions (80% N_2/20% CO_2 headspace) at 37°C using stoppered 134 mL serum vials.

Northern Blot RNA purification and deacylation

M. acetivorans was grown to exponential phase in both conditions (n = 4 biological replicates for each condition) and harvested by centrifugation (12K × g, 2 minutes, 4°C) over ice. Cell pellets were treated in parallel and resuspended in 1 mL of TRIreagent (Zymo), flash frozen in liquid nitrogen, and lysed by bead beating (Mini-G HG-400, Cole-Parmer) 3 times for 30 seconds at 1500 RPM using 2.4 mm metal beads (Cole-Parmer). Total RNA was isolated by adding 200 μL of chloroform and centrifuged (21K × g, 15 minutes, 4°C). The aqueous phase was mixed with an equal volume of 100% isopropanol and RNA was pelleted immediately (21K × g, 20 minutes, 4°C). The RNA pellet was washed with 500 μL of cold 70% ethanol, centrifuged (21K × g, 10 minutes, 4°C), and resuspended in 10 μL of 10 mM sodium acetate pH 4.5.

The deacylation protocol was adapted from previously described methods (2). 15 μg of RNA from each sample was incubated at 42°C for 90 minutes in 1mM EDTA and 100 mM Tris pH 9 in a final volume of 50 μL. The reaction is quenched by adding 5 μL of 3M sodium acetate pH 5.2. Deacylated RNA was precipitated by adding 50 μL of 100% isopropanol and incubated at -20°C for one hour. Precipitated RNA was centrifuged (21K × g, 20 minutes, 4°C) and the pellet was washed with 1 mL of cold 70% ethanol. The deacylated RNA pellet was resuspended in 10 μL of 10 mM sodium acetate pH 4.5.

For the carbon source switchover assay, 10 mL of cells were grown to exponential phase (OD_{600} = 0.6-0.8), and the culture was centrifuged at 5000 × g for 5 minutes at 4°C. The supernatant was removed and the cells were resuspended in 1 mL of basal growth media with no carbon source. 500 μL of the resuspended culture was put into a new 10 mL tube with either methanol or TMA. These cultures were grown at 37°C for 18 hours, then centrifuged for 1 minute at 4°C. The pellet was then resuspended in 1 mL Trizol and processed identically to the initial northern blot assays.

tRNA Northern Blot

The northern blot protocol was performed as previously described (3). To assess tRNA charging, 2 μg of total RNA and deacylated RNA samples were run on a 12% denaturing polyacrylamide acid gel containing 8 M urea and 0.1 M sodium acetate pH 5.2 with a custom loading dye (8 M urea, 10 mM sodium acetate pH 5.0, 1 mM EDTA pH 8, 0.01% bromophenol blue) and running buffer (100 mM sodium acetate pH 5.0, 1 mM EDTA pH 8) for 5.5 hours at 100 V and 4°C. RNA was transferred to an Amersham Hybond™ -N+ (Cytiva) for 30 minutes at 0.6 A on the Invitrogen Power Blotter. The membrane was dried for 5 minutes at 50°C and crosslinked for 90 seconds (254 nM 1200 mJ). The membrane was prehybridized for 2 hours at 42°C in 10 mL of hybridization buffer (G-Biosciences). Hybridization buffer was exchanged and 1 μL of each 10 μM IRDye-labeled oligonucleotide probe (IDT) specific for pyrrolysine tRNA

(IR700-5’-CTACATGATCAGGTTTCC-3’) and alanine tRNA (IR800-5’-CCTGATCTACGAGCCC-3’) was added and incubated overnight in a hybridization oven at 42°C. After hybridization, the membrane was washed for 10 minutes with 2× SCC + 0.1% SDS followed by an additional wash with 1× SCC + 0.1% SDS and imaged on the Azure 600 imaging system (Azure Biosystems).

The tRNA fragment northern blots were performed as previously described with specific changes (4). Small RNA was separated from total RNA using the Monarch Spin RNA Cleanup Kit (50 µg) (NEB) following manufacturer’s instructions for size-based separation. RNA was precipitated for 1 hour at -20°C with equal parts isopropanol. Small RNA was pelleted by centrifugation (21K × g, 20 minutes, 4°C) and washed with 500 µL of cold 70% ethanol, centrifuged (21K × g, 10 minutes, 4°C), and resuspended in 5 µL of nuclease free water. RNA was heated at 80°C for three minutes and immediately placed on ice prior to separation on a 15% Urea TBE polyacrylamide gel (200V, 1 hour). RNA was transferred for 7 minutes at 1.6 A.

<u>RT-qPCR</u>

Total RNA was isolated from *M. acetivorans* lysate (n = 3 biological replicates for each condition) obtained from ribosome profiling using the Direct-zol RNA MicroPrep Kit TriReagent with TRIreagent (Zymo) following manufacturer’s instructions, including DNase I treatment. The RNA was then diluted to 100 ng/µL and RT-qPCR was performed on the Applied Biosystems QuantStudio 6 in 20 µL reactions using the Luna Universal Probe One-Step RT-qPCR Kit (NEB) with 1 µL of diluted total RNA, 0.8 µL of each primer, and 0.4 µL of each probe (Table S1). Thermal cycling conditions were: Reverse transcription at 55°C for 10 minutes, initial denaturation at 95°C for 1 minute, and 40 cycles of denaturation at 95°C for 10 seconds and extension at 60˚C for 1 minute. Relative expression was calculated using the ΔΔCt method using reference genes as the internal control (5).

<u>RNA-Seq</u>

M. acetivorans was grown (n = 2 biological replicates for each condition), harvested, and lysed as previously described. The RNA was extracted from the lysate using the Direct-Zol RNA MiniPrep Kit (Zymo) with DNase I treatment according to the manufacturer’s instructions. Purified total RNA was rRNA depleted using a riboPOOLs Pan-Archaea rRNA Depletion kit (siTOOLS). RNA libraries were prepared using the NEBNext Ultra II Directional RNA Library Prep kit. Libraries were quantified with a Qubit (Invitrogen), pooled to 10 nM and sequenced by SeqCenter (Pittsburgh, PA) using the Illumina NextSeq 2000 with 2×151bp reads and 10% PhiX.

<u>Ribosome Profiling</u>

M. acetivorans was grown as previously described (n = 3 biological replicates for each condition) and harvested by centrifugation (12K × g, 2 minutes, 4°C) over ice and the pellets were then removed from the bottle using a metal scoop that was pre-cooled with liquid nitrogen and immediately frozen in liquid nitrogen until processing. Frozen cell pellets were lysed by bead (200 µm zirconium) beating with Mini-G HG-400,(Cole-Parmer) 5 times for 1 minute at 1500 RPM with 1 minute cooling on ice between rounds in 1 mL 1x lysis buffer (20 mM Tris pH 8, 100 mM KCl, 50 mM $MgCl_2$, 0.5% Triton X-100, 2 mM DTT, 100 U/mL DNase I (Zymo)). 50 µL of lysate was saved for RT-qPCR. Lysate was clarified by centrifugation (10K × g, 10 minutes, 4°C) and diluted 7.5 mL using 1× polysome buffer (20 mM Tris pH 8, 100 mM KCl, 50 mM $MgCl_2$) and layered over a 2.5 mL 60% sucrose cushion (20 mM Tris pH 8, 100 mM KCl, 50 mM $MgCl_2$, 60% sucrose) then pelleted in the Ti70.1 rotor for 2 hours at 70K RPM and 4°C in the Optima XPN-90 Ultracentrifuge (Beckman Coulter). The ribosome pellets were resuspended in

800 μL of digestion buffer (20 mM Tris pH 8, 10 mM $MgCl_2$, 100 mM KCl) with stir bars for 20 minutes at 4°C. Each sample was split and treated with 5 U/μg RNase I_f (NEB) and 10 U/μg P1 (NEB). All samples were treated for 1 hour at room temperature then the reaction was quenched by adding 5 μL of RNase Inhibitor. Spin-X UF 500 100K MWCO PES 500 μL columns (Corning) were primed with 500 μL of 1× polysome buffer and centrifuged (12K × g, 7 minutes, 4°C). The treated samples were then added to the column and centrifuged (12K × g, 9 minutes, 4°C) then washed with 500 μL of 1× polysome buffer and centrifuged again (12K × g, 9 minutes, 4°C). The ribosome protected fragments were isolated by phenol chloroform extraction from 100 μL of retentate followed by the Monarch Spin RNA Cleanup Kit 50 μg (NEB) eluting in 50 μL. The ends of the RNase I treated samples were repaired using T4 PNK (NEB) for 30 minutes at 37°C. Both the P1 and the RNase I treated samples were isopropanol precipitated and resuspended in 6 μL of RNase-free water, denatured for 3 minutes at 80°C and separated on a 15% 8 M urea polyacrylamide gel for 80 minutes at 200 V with the ZR small RNA ladder (Zymo) and Low-Range ssRNA ladder (NEB). The monosome (25-29 nt) and disome (45-60 nt) fragments were excised from the gel after being visualized with SYBR Gold (Thermo), crushed, and extracted by adding 500 uL of RNA extraction buffer (0.25% SDS, 10 mM EDTA, 0.3 M sodium acetate). The crushed gel slurry was incubated at 65°C for 10 minutes, transferred to liquid nitrogen, then thawed at 65°C prior to being incubated overnight on rotator at room temperature. The gel was pelleted by centrifugation (10K × g, 10 minutes, 4°C) and the supernatant was precipitated as previously described. The RNA pellet was dried at 37°C for 10 minutes then resuspended in 11 μL of nuclease free water. The resuspended gel pellet was centrifuged again (10K × g, 10 minutes, 4°C) to remove any residual gel and the top 10 μL was used. 5 ng of RNA was used for the NEB LB Small RNA Library Prep Kit with LV indexes and libraries were prepared following the manufacturer's instructions. 5 μL of 6× DNA loading dye (NEB) was added to the 15 μL of cleaned up library and ran on a 6% native polyacrylamide gel for 60 minutes at 120V. The DNA was cut out of the gel, crushed, and 500 μL of DNA Gel Elution Buffer was added (NEB) and was incubated overnight at room temperature to elute. The DNA was isolated as previously described and resuspended in 8 μL of nuclease free water. Libraries were quantified using Qubit (Invitrogen) and quality analyzed using a TapeStation 4150 (Agilent). The libraries were pooled to 15 nM and sequenced by SeqCenter (Pittsburgh, PA) using the Illumina NovaSeq X with 2×151bp reads and 25% PhiX.

Small RNA Sequencing

M. acetivorans was grown (n = 2 biological replicates for each condition) and harvested as previously described. The cell pellets were resuspended in 400 μL of TRIreagent (Zymo) and the RNA was extracted using standard phenol chloroform. The RNA was treated with DNase I and isolated using the Direct-zol™ RNA MiniPrep with TriReagent kit (Zymo) following manufacturer's instructions. The small RNA was then separated from total RNA using the Monarch Spin RNA Cleanup Kit (50 μg) (NEB) following manufacturer's instructions for size-based separation. The ends of the small RNA were repaired as previously described and cleaned up using the Monarch Spin RNA Cleanup Kit (10 μg) (NEB). Purified small RNA was then run on a 15% 8 M urea polyacrylamide gel for 45 minutes at 300V with the Low-Range ssRNA Ladder (NEB). RNA 15-70 nt was cut out of the gel, crushed, extracted, and precipitated as previously described. 20 ng of RNA was used for the TruSeq Small RNA Library Prep Kit (Illumina) and libraries were prepared following the manufacturer's instructions. Libraries were quantified using Qubit (Invitrogen) and pooled to 10 nM and sequenced by SeqCenter (Pittsburgh, PA) using the Illumina NextSeq 2000 with 1×100bp reads and 25% PhiX.

Preprocessing of sequencing samples

Adapters were removed using cutadapt v5.2, allowing for 1 mismatch and keeping only reads with Q ≥ 30. Reads for ribo-seq were then filtered by length (min = 12 nt, max = 35 nt). Non-coding RNA removal (for RNA-seq and ribo-seq samples) was performed using Bowtie2 v2.5.4 to remove reads that end-to-end mapped to regions identified as non-coding (annotation: GCF_000007345.1-RS_2025_11_20) in the *M. acetivorans* C2A (NC_003552.1) genome. Reads that passed filter were then mapped using Bowtie2 to the reference genome, allowing for 1 mismatch and discarding any multimapping reads. The resulting files were sorted and indexed using samtools v1.22.1 and counted using featureCounts (subread v2.1.1).

Differential expression analysis

Differential expression analysis was performed with DESeq2 using a negative binomial generalized linear model and Wald tests for the specified contrast (6). Log_2FC was shrunken using lfcShrink with the apeglm method to provide stabilized effect-size estimates for ranking and visualization. P-values from Wald tests were adjusted for multiple testing using the Benjamini–Hochberg false discovery rate procedure. Genes were considered differentially expressed if $p_{adj} \leq 0.05$ and $|Log_2FC| \geq 1$.

Ribosome profiling data analysis

Ribosome profiling data were analyzed using a combination of previously published methods (7-12).To adapt these protocols to our dataset, we used Microsoft 365 Copilot as a coding assistant to generate initial drafts of selected scripts (e.g., boilerplate structure, helper functions, and visualization routines) based on author-specified requirements and peer-reviewed reference code. Copilot assistance was limited to code scaffolding for the offset sweep, pause score, and collision index pipelines; analytic decisions, parameter selection, and interpretation were determined by the authors. All generated code underwent manual review, revision, and testing, and was validated against published datasets and expected outputs (8, 13). The authors take full responsibility for the final codebase and all analyses.

Periodicity offset sweep

To identify the read length and end-based offset that maximized 3-nt periodicity, we pooled uniquely mapped ribosome-protected fragments and evaluated candidate read lengths and offsets. For each read length *L* and offset *O*, we defined an end coordinate for each primary alignment (3′-end by default), then inferred a P-site position by shifting that end coordinate by *O* nucleotides in a strand-aware manner. P-site positions were retained only if they fell within annotated coding sequences (CDS) from the reference annotation (GCF_000007345.1-RS_2025_11_20), with CDS intervals extended by 3 nt to include the stop codon. For each (*L*, *O*) pair, we assigned each retained P-site to a reading frame relative to the CDS start (frame = 0/1/2, strand-aware) and calculated the fraction of CDS-assigned reads in frame 0. The optimal (*L*, *O*) combination was selected as the pair that maximized the frame-0 fraction.

Pause score

We calculated the pause score for each condition by aggregating the reads from biological replicates (n = 3) grown in either MeOH or TMA. The A-site codon was identified by determining P-site coordinate using the 27 nt read length with an offset of 15 nt and shifting it by +3 nt in the CDS frame. The codon sequence was assigned from the extracted reference annotation (GCF_000007345.1-RS_2025_11_20) with strand-aware orientation. The final 5 codons (15 nt) and any in-frame premature TAA and TGA stop codons were excluded from our both our

observed read assignment and expected codon abundance calculations to prevent noise due to ribosome queueing caused by terminating ribosomes. Biological replicates were processed independently then summarized as the mean pause score across replicates. Variability was calculated as standard error of the mean.

Two complementary pause score normalization metrics were computed from A-site assignments:

1. Gene normalized, site-weighted pause score: Within each gene, the mean ribosome occupancy was computed as total A-site assigned reads divided by the gene length to control for total ribosome density on the gene. Codon-specific density was computed as A-site assigned reads of a specific codon divided by the number of times the codon appeared in the gene. A per-gene pause score was defined as the ratio of the codon-specific ribosome density divided by the mean gene density. The pause scores were then weighted so that each occurrence of a codon in contributed equally to the pause score.
2. Global normalization, expression weighted: The expected A-site codon frequency was calculated using RNA-seq data in matching conditions to remove bias from highly expressed genes using mean transcript abundance (CPM). The global pause score was calculated as a ratio of observed A-site frequency to expected codon frequency.

Collision Index

The collision index was calculated by taking the ratio of the pause score (paired by condition and normalization method) from monosome reads (length = 27 nt, offset = 15 nt) and disome (length = 54 nt and 57 nt, offset = 15 nt). We combined the 54 nt and 57 nt lengths for disomes to increase our signal and because both read lengths had robust 0-frame read percentages. To prevent dividing by 0, we added a pseudocount (1×10^{-9}) to the monosome and disome pause scores. Variance was calculated by standard error of the mean.

Supplemental figures

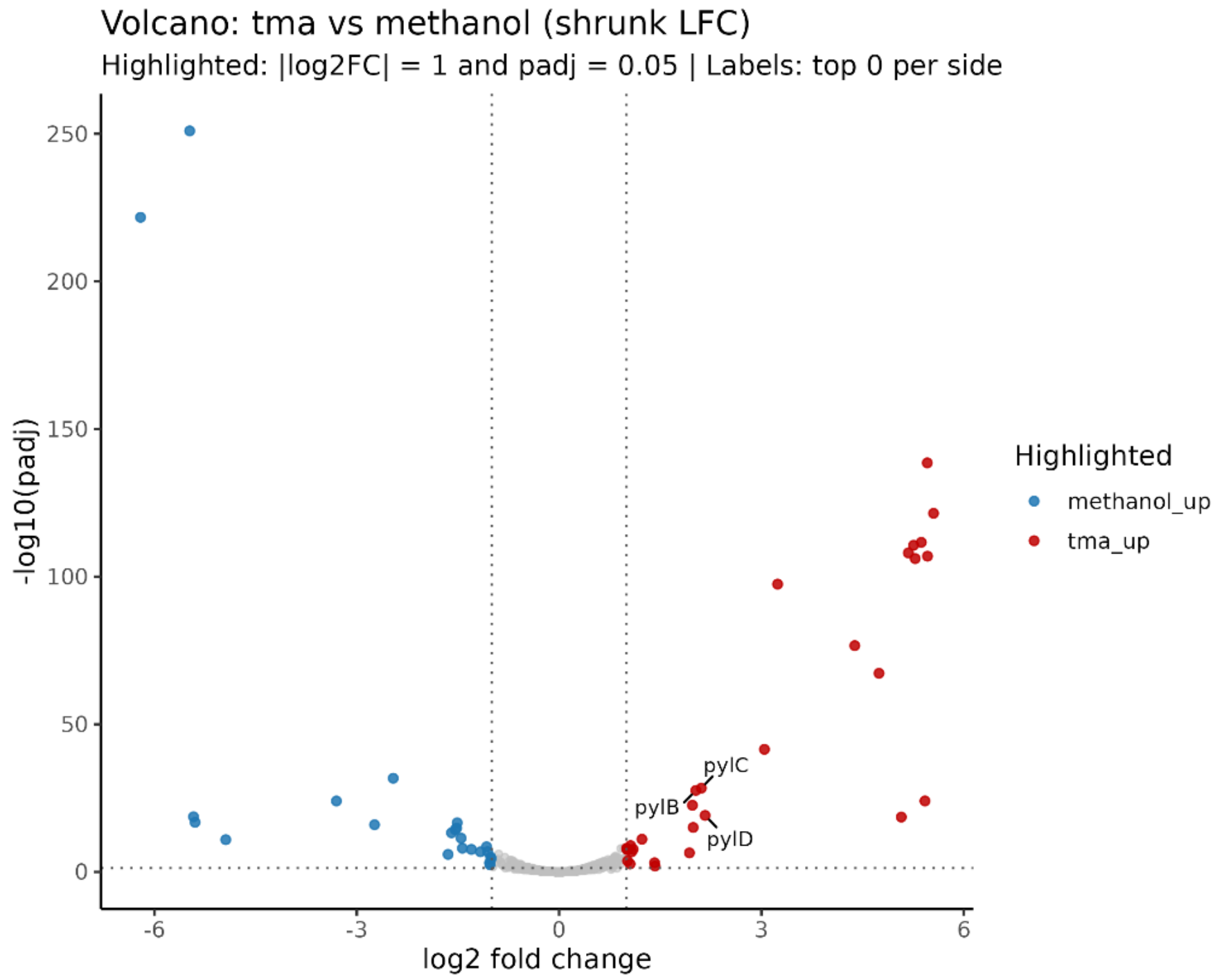

Supplementary figure 1: Volcano plot of RNA-seq data. Upregulated in TMA conditions is highlighted on the right in red and upregulated in methanol conditions is highlighted in blue on the left.

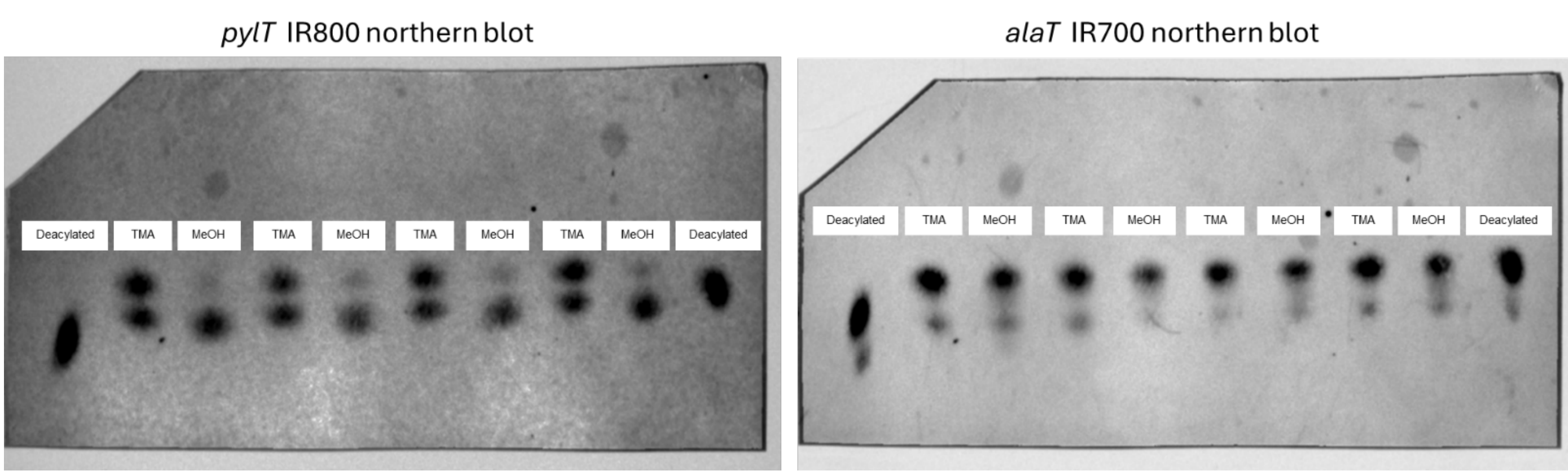

Supplementary figure 2: Full uncropped northern blots used for **Figure 1B** and calculation for **Figure 1C**.

pylT IR800 northern blot

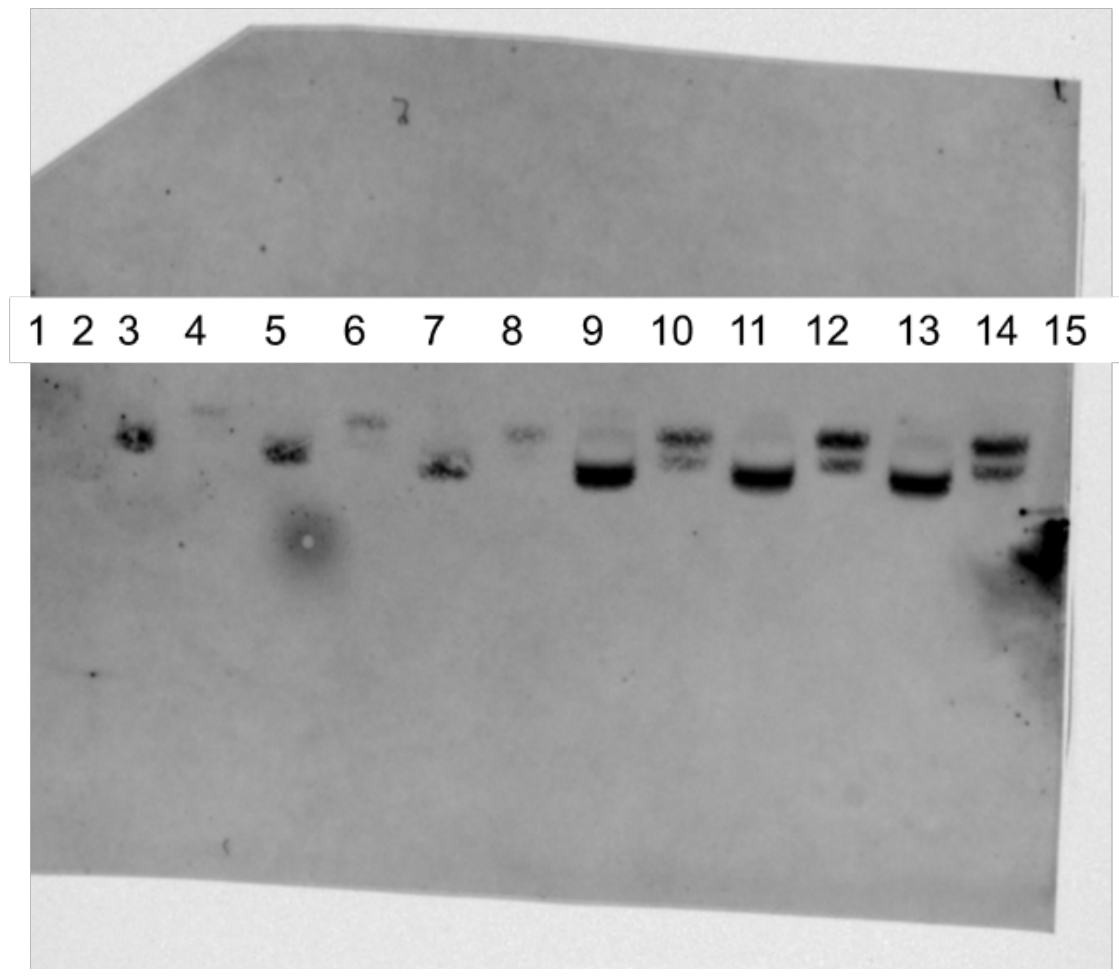

alaT IR700 northern blot

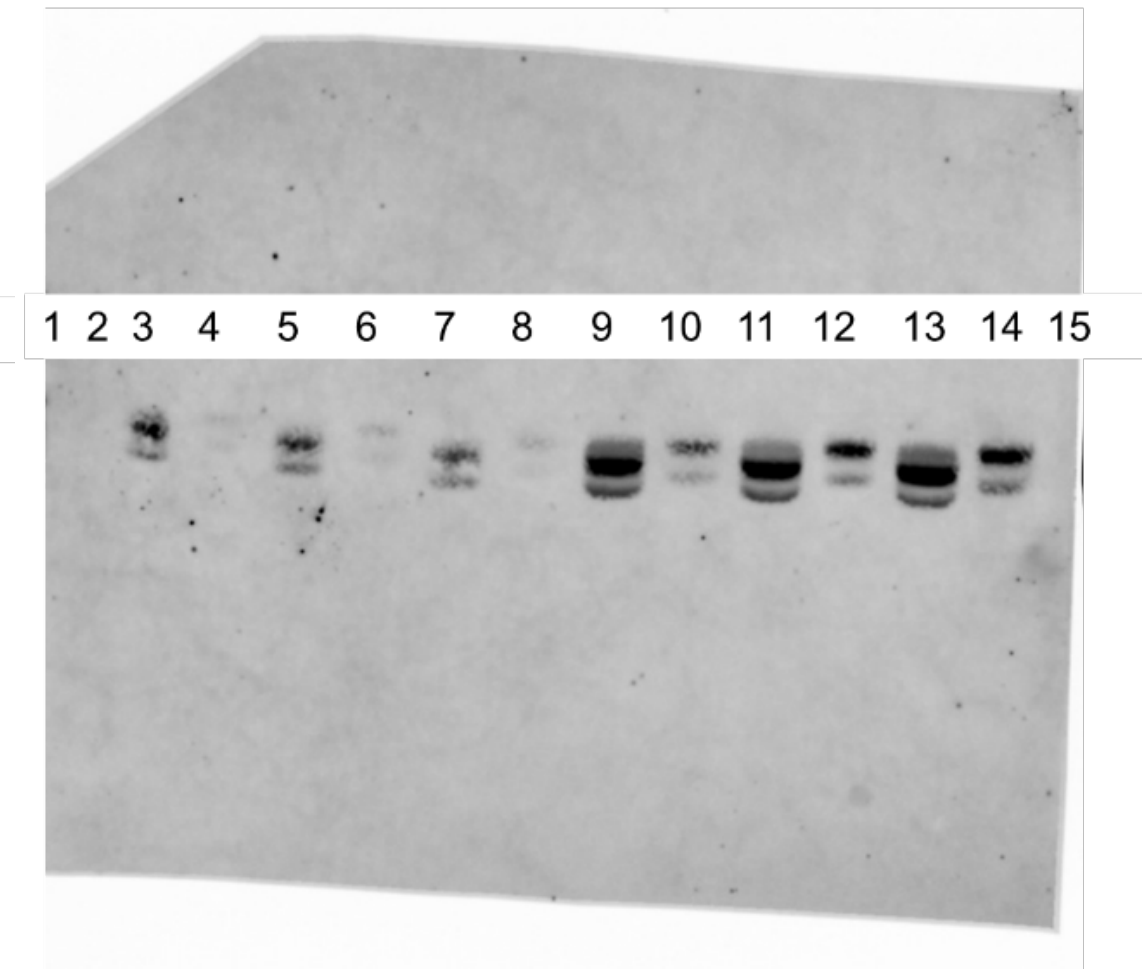

Supplementary figure 3: Full uncropped northern blots used for **Figure 1E** and calculation for **Figure 1F**. Only lanes 9-14 were used. Lanes 1, 2 and 15 are intentionally blank. Lanes 3, 5, 9, 11, and 13 are switchovers from methanol to methanol after 18 hours. Lanes 4, 6, 8, 10, 12, and 14 are switchovers from methanol to TMA after 18 hours.

Methanol Pyl IR700

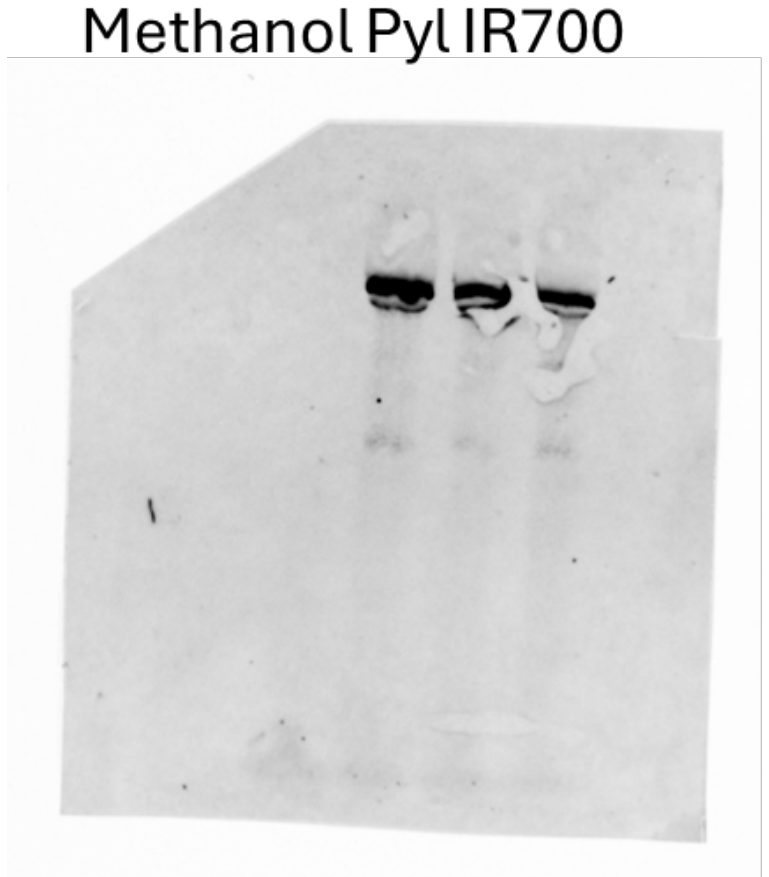

TMA Pyl IR700

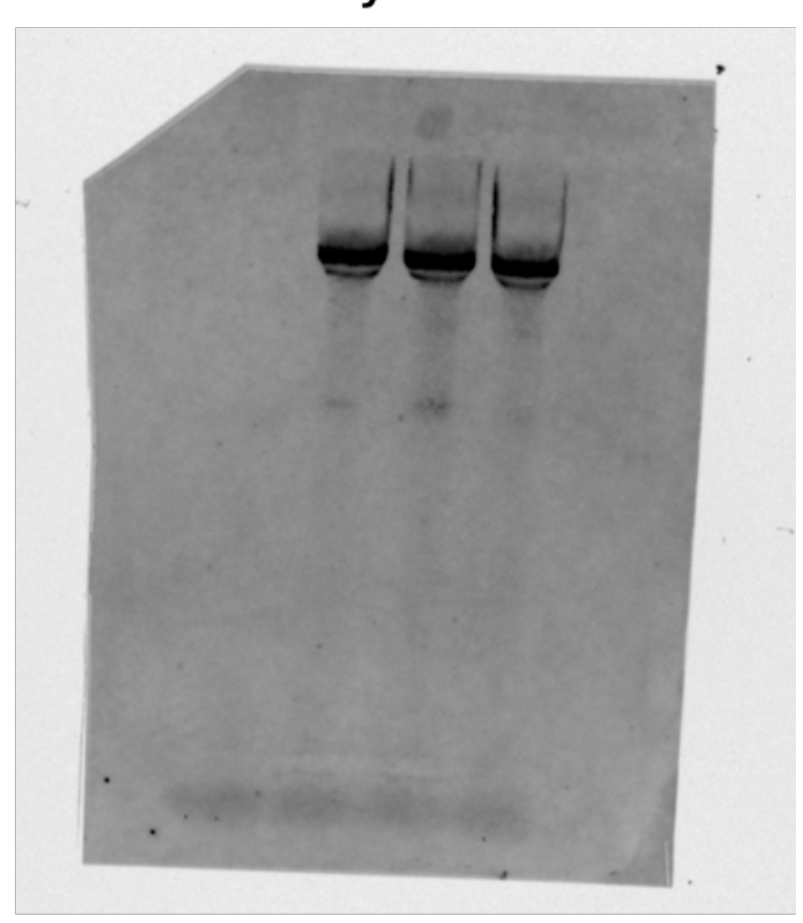

Methanol Ala IR800

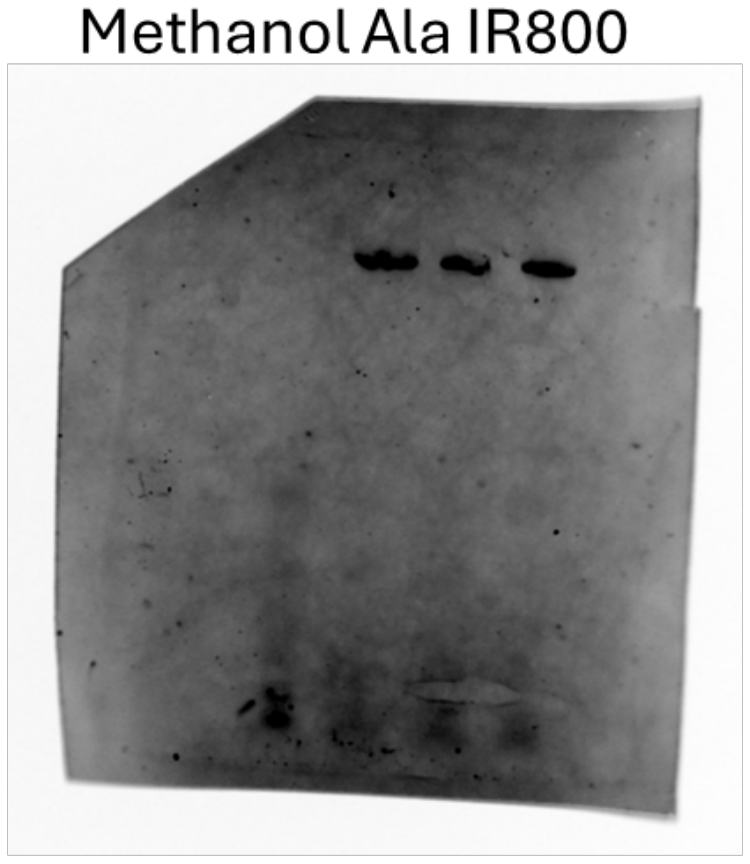

TMA Ala IR800

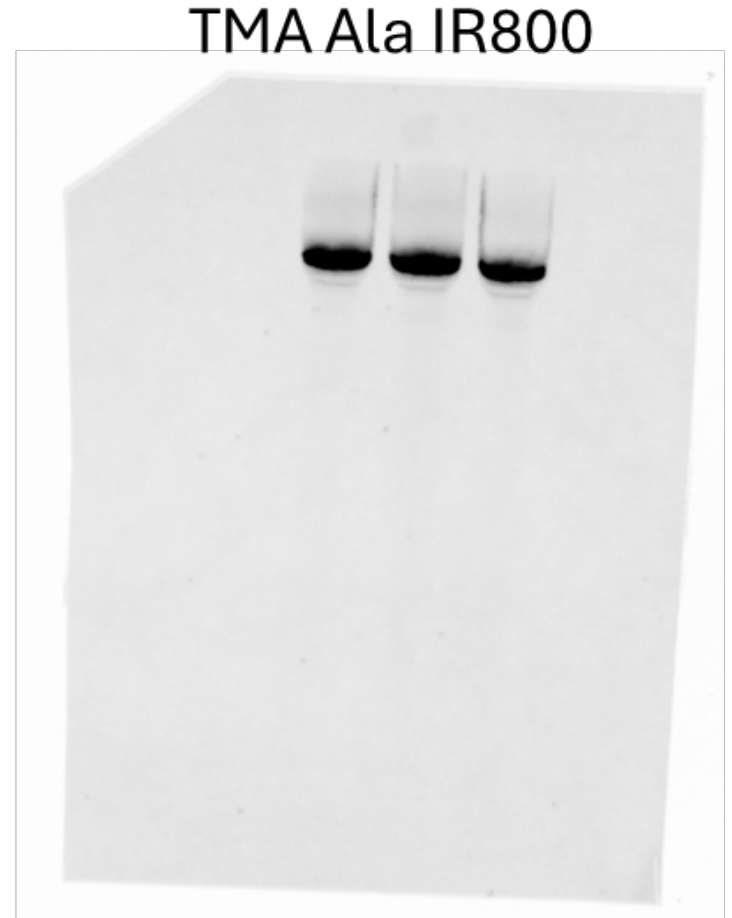

Supplementary figure 4: Full uncropped northern blots used for **Figure 1H**.

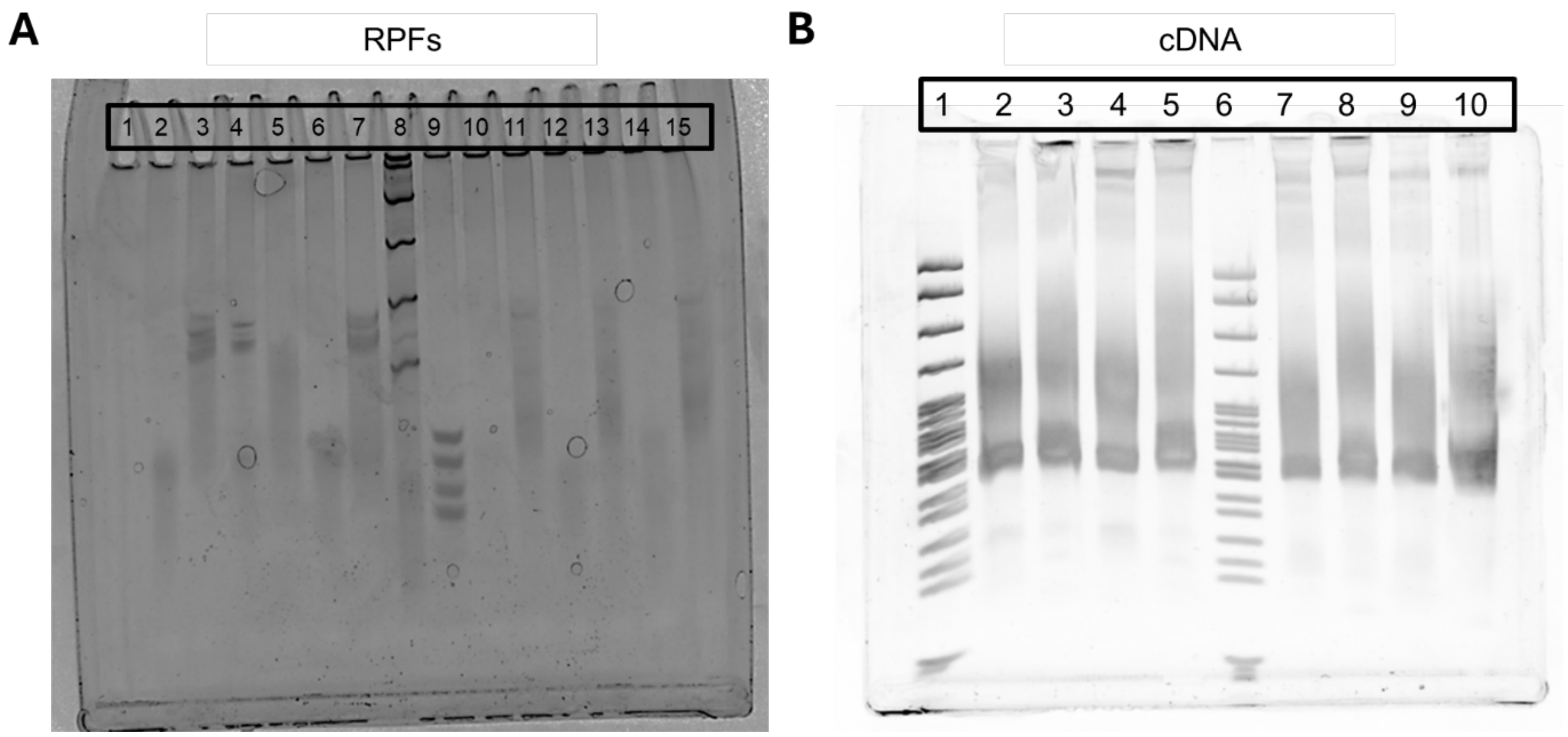

Supplementary figure 5. Generation of RPF libraries. (**A**) Quality control gel of size-selected RPFs, 15% 8M urea-PAGE. Methanol monosome (2,4,6), disome (3,5,7), TMA monosome (10,12,14) and disome (11,13,15). Lane 1 is left intentionally blank. Lane 8 is NEB Low-Range ssRNA ladder and lane 9 is ZR small RNA ladder. (**B**) Representative cDNA library gel, 6% PAGE. Methanol monosome (2,4) and disome (3,5) with TMA monosome (7,9) and disome (8,10). Lanes 1 and 6 are NEB pBR322 DNA-MspI Digest ladders.

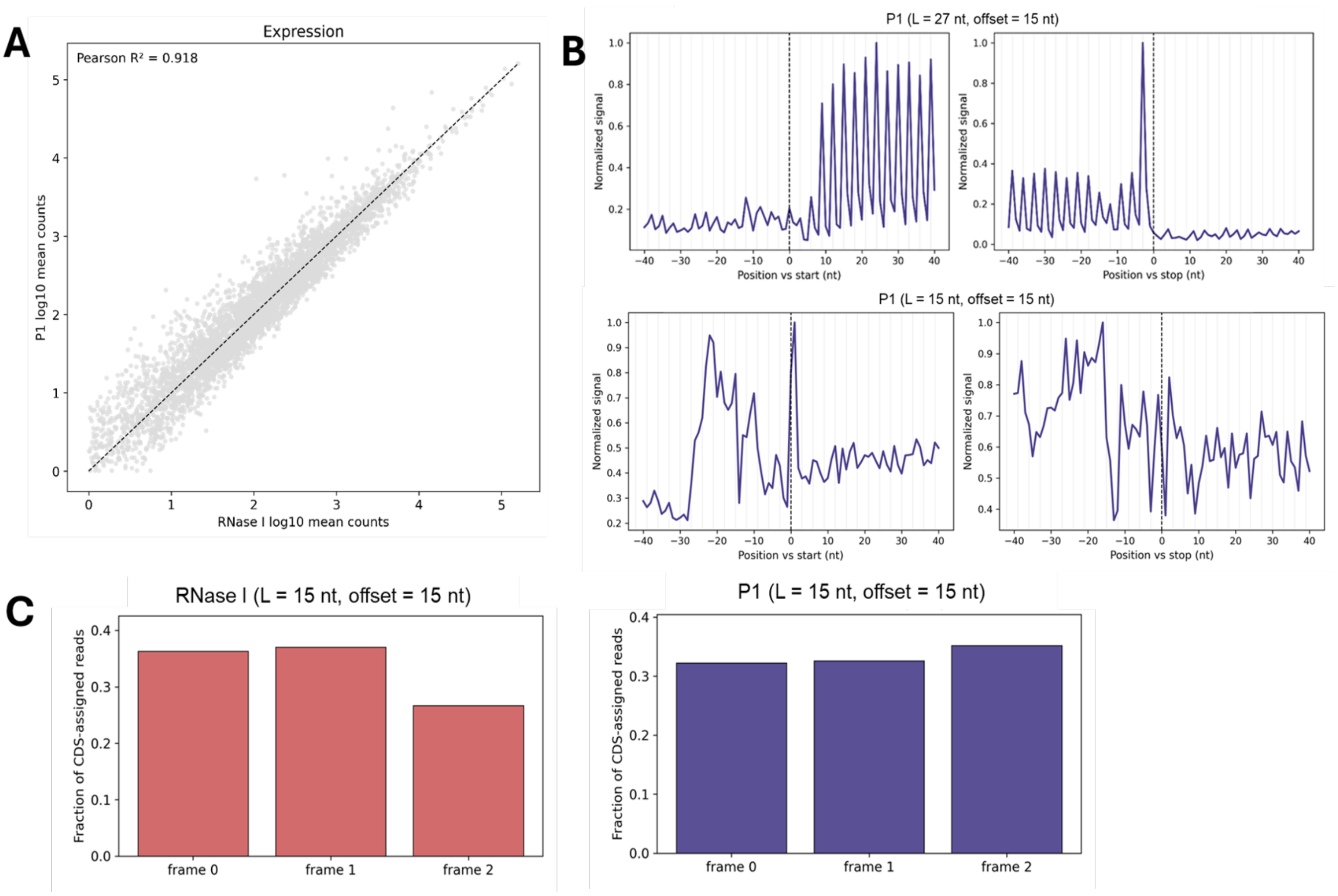

Supplementary figure 6. Further analysis of P1 ribosome profiling dataset. (**A**) Correlation of RPFs generated by RNase I and nuclease P1 (n = 3 biological replicates for per nuclease). (**B**) Metagene analysis of P1-generated RPFs. (**C**) 3-nt periodicity of 15 nt reads for both RNase I (left) and P1 (right).

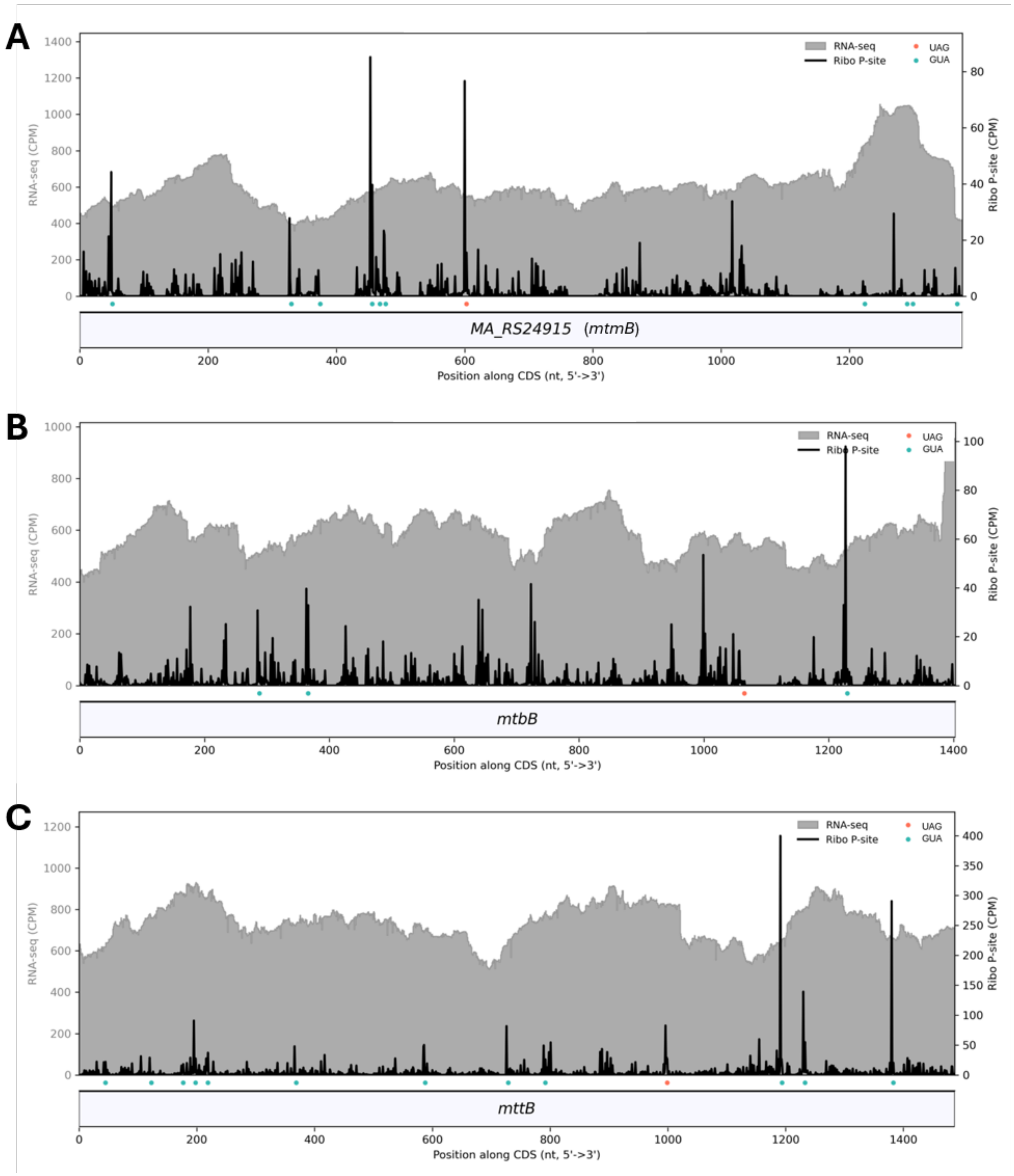

Supplementary figure 7. Representative coverage graph for the (A) *mtmB* (B) *mtbB* and (C) *mttB* displaying the ribosome P-site density in black and RNA-seq coverage of each gene displayed in grey in the background. GUA codons are denoted by blue points and UAG are denoted by red dots.

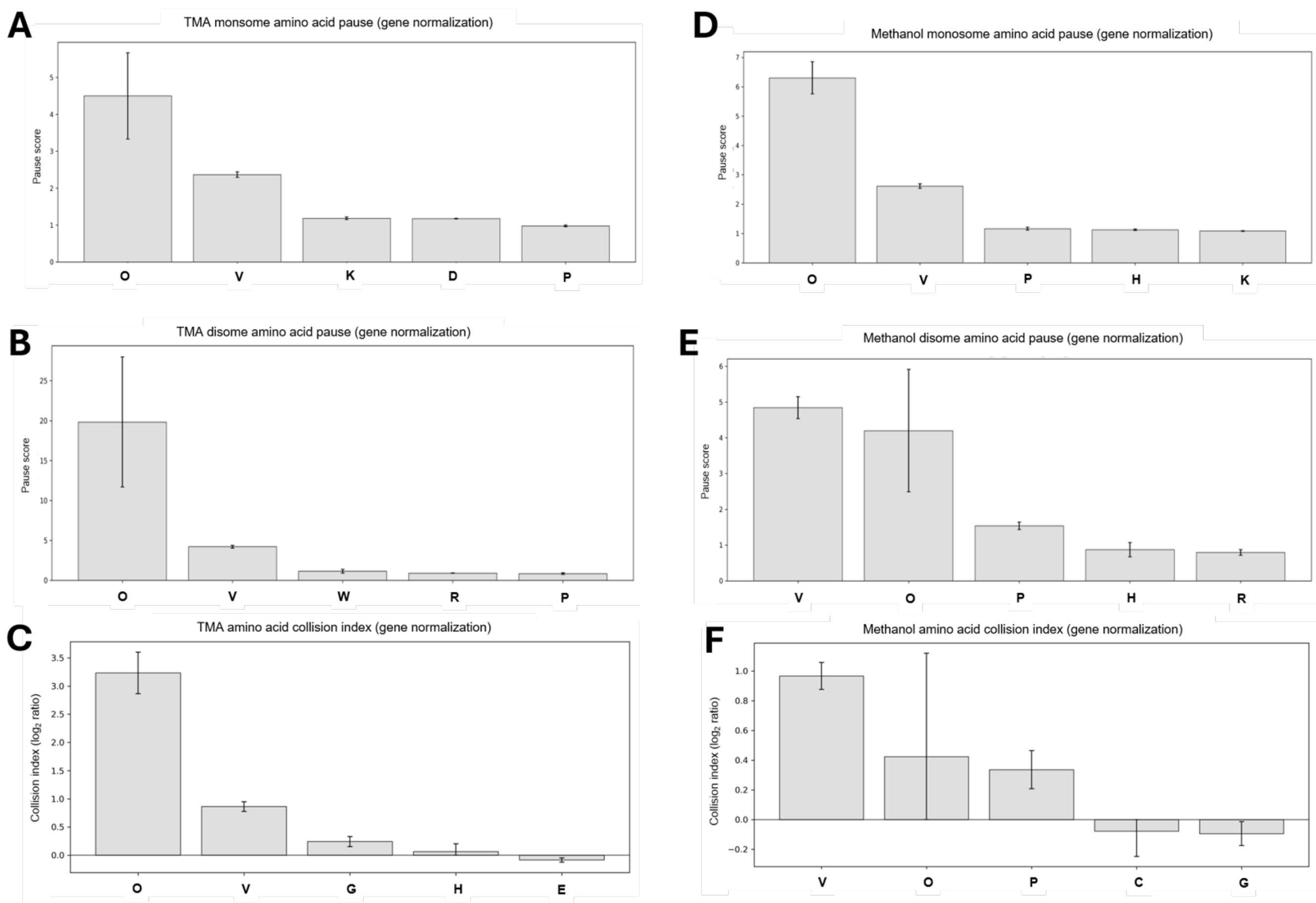

Supplementary figure 8. Pause score and collision index for monosomes and disomes in both conditions (amino acid resolution). Top five amino acid pause scores with gene normalization for RNase I treated TMA monosome (**A**) and disome (**B**) samples. The x-axis displays the amino acid, and the pause score is displayed on the y-axis. Monosome samples are 27 nt and disome samples use lengths ranging from 54-57 nt. (**C**) Top five amino acid collision indexes using $\log_2$ ratio comparing Rnase I treated TMA monosome and disome samples. The x-axis displays the amino acid, and the collision index is displayed on the y-axis. Top five amino acid pause scores with gene normalization for Rnase I treated MeOH monosome (**D**) and disome (**E**) samples. The x-axis displays the amino acid, and the pause score is displayed on the y-axis. Monosome samples are 27 nt and disome samples use lengths ranging from 54-57 nt. (**F**) Top five amino acid collision indexes using $\log_2$ ratio comparing Rnase I treated MeOH monosome and disome samples. The x-axis displays the amino acid, and the collision index is displayed on the y-axis.

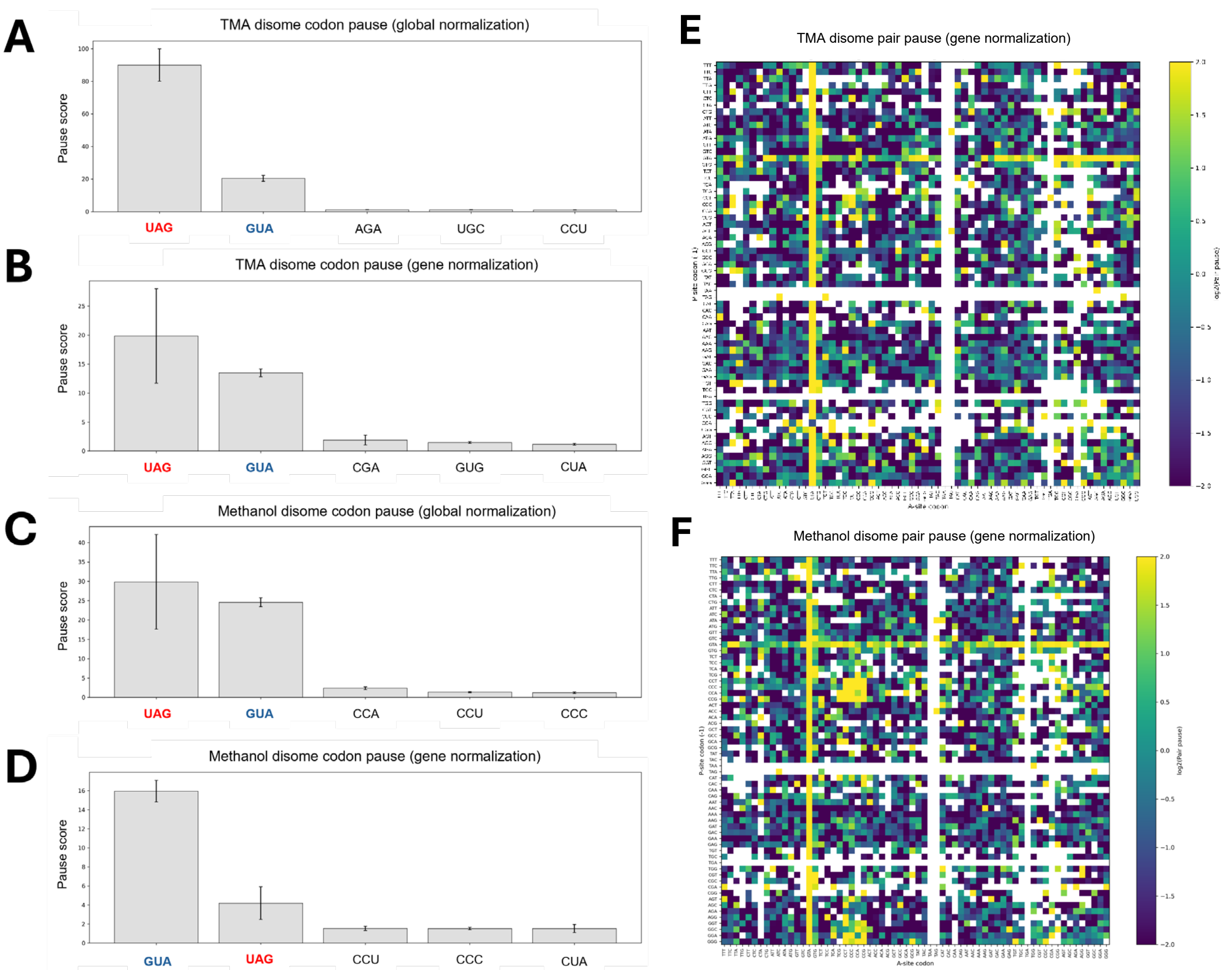

Supplementary figure 9. Disome pause score and pair pause analysis. Top five codon pauses for TMA (**A,B**) and MeOH (**C,D**) disome footprints (54, 57 nt) using the global ratio (**A,C**) and gene normalized (**B,D**) methods. Pause score is calculated by A-site coverage of each specific codon. Pair pause heat map showing the TMA (**E**) and MeOH (**F**) disome pause score of the A-site codon (x-axis) dependent on the P-site codon (y-axis). The white squares with a red X are for pairs with read counts below the threshold.

Table S1. Synthetic oligonucleotides used in this study

Primer name	Sequence (5’-3’)	Probe
pylT IRDYE northern	[IR700]CTACATGATCAGGTTTCC	
alaT IRDYE northern	[IR800]CCTGATCTACGAGCCC	
pylT RT-qPCR F	GGAAACCTGATCATGTAGATCGAA	
pylT RT-qPCR R	GCGGAAACCCCGGGAA	
pylB RT-qPCR F	AGCCCGAGTTCGTTCTTTAC	
pylB RT-qPCR R	CCATGGGAGAAGACCCTTATTAC	
pylC RT-qPCR F	ATCCGGTAAGCCTCGAAATC	
pylC RT-qPCR R	GACGCCATACTCCCTGTAAAT	
rpoB RT-qPCR F	GTAAACGGCATGTCAGCAATAG	
rpoB RT-qPCR R	AGTAAAGGTAAGGGACGAAAGG	
alaT RT-qPCR F	GGCTCGTAGATCAGGGGAAG	
alaT RT-qPCR R	GGACTCGGCGGGATTTGAA	
pylT RT-qPCR probe	TCTAAATCCGTTCAGCCGGGTTA	Int ZEN™<br>5' HEX<br>3' Iowa Black™ FQ
pylB RT-qPCR probe	ATCTGGACAAGCTCAACGAAGCGG	Int ZEN™<br>5' 6-FAM<br>3' Iowa Black™ FQ
pylC RT-qPCR probe	AGGACAGGGCAGGAGAATTCCTCT	Int ZEN™<br>5' HEX<br>3' Iowa Black™ FQ
rpoB RT-qPCR probe	CCGTGTCTGGAAGCGAACTTGTCT	Int ZEN™<br>5' Cy5<br>3' Iowa Black™ FQ
alaT RT-qPCR probe	ATCGTTACGTTCGCAACGTAAAGGCCGCGG	Int ZEN™<br>5' 6-FAM<br>3' Iowa Black™ FQ

Legends for Datasets S1-S3

Dataset S1. RNA-seq differential expression results comparing methanol-grown and trimethylamine-grown samples.

Dataset S2. Ribosome profiling differential expression results comparing methanol-grown and trimethylamine-grown samples.

Dataset S3. Small RNA-seq differential expression results comparing methanol-grown and trimethylamine-grown samples.

SI References

1. J. E. Galagan *et al.*, The Genome of <i>M. acetivorans</i> Reveals Extensive Metabolic and Physiological Diversity. *Genome Research* **12**, 532-542 (2002).
2. L. Yin, C. S. Harwood, Charging State Analysis of Transfer RNA from an α-proteobacterium. *Bio Protoc* **10**, e3834 (2020).
3. B. D. Janssen, E. J. Diner, C. S. Hayes, "Analysis of Aminoacyl- and Peptidyl-tRNAs by Gel Electrophoresis". (Humana Press, 2012), 10.1007/978-1-61779-949-5_19, pp. 291-309.
4. R. Heyer *et al.*, High throughput sequencing reveals a plethora of small RNAs including tRNA derived fragments inHaloferax volcanii. *RNA Biology* **9**, 1011-1018 (2012).
5. K. J. Livak, T. D. Schmittgen, Analysis of Relative Gene Expression Data Using Real-Time Quantitative PCR and the 2−ΔΔCT Method. *Methods* **25**, 402-408 (2001).
6. M. I. Love, W. Huber, S. Anders, Moderated estimation of fold change and dispersion for RNA-seq data with DESeq2. *Genome Biology* **15** (2014).
7. F. Mohammad, R. Green, A. R. Buskirk, A systematically-revised ribosome profiling method for bacteria reveals pauses at single-codon resolution. *eLife* **8** (2019).
8. D. R. Gelsinger *et al.*, Ribosome profiling in archaea reveals leaderless translation, novel translational initiation sites, and ribosome pausing at single codon resolution. *Nucleic Acids Research* **48**, 5201-5216 (2020).
9. L. Ferguson *et al.*, Streamlined and sensitive mono- and di-ribosome profiling in yeast and human cells. *Nature Methods* **20**, 1704-1715 (2023).
10. P. Han *et al.*, Genome-wide Survey of Ribosome Collision. *Cell Reports* **31**, 107610 (2020).
11. A. B. Arpat *et al.*, Transcriptome-wide sites of collided ribosomes reveal principles of translational pausing. *Genome Research* **30**, 985-999 (2020).
12. T. Zhao *et al.*, Disome-seq reveals widespread ribosome collisions that promote cotranslational protein folding. *Genome Biology* **22** (2021).
13. M. A. Tufail *et al.*, Uncovering the small proteome of Methanosarcina mazei using Ribo-seq and peptidomics under different nitrogen conditions. *Nature Communications* **15** (2024).
